# PU.1-c-Jun interaction is crucial for PU.1 function in myeloid development

**DOI:** 10.1038/s42003-022-03888-7

**Published:** 2022-09-14

**Authors:** Xinhui Zhao, Boris Bartholdy, Yukiya Yamamoto, Erica K. Evans, Meritxell Alberich-Jordà, Philipp B. Staber, Touati Benoukraf, Pu Zhang, Junyan Zhang, Bon Q. Trinh, John D. Crispino, Trang Hoang, Mahmoud A. Bassal, Daniel G. Tenen

**Affiliations:** 1 Harvard Stem Cell Institute, Harvard Medical School, Boston, MA 02115 USA; 2 Union Hospital, Tongji Medical College, Huazhong University of Science and Technology, Wuhan, Hubei China; 3 Albert Einstein College of Medicine, New York, NY USA; 4 Department of Biomedical Sciences, College of Life and Health Sciences, Chubu University, Kasugai, Aichi Japan; 5 Department of Hematology-oncology, Institute of Molecular Genetics of the Czech Academy of Sciences, Vídeňská, Prague Czech Republic; 6 Childhood Leukemia Investigation Prague, Department of Pediatric Haematology and Oncology, 2nd Faculty of Medicine, Charles University in Prague, University Hospital Motol, Videnska, Czech Republic; 7 Department of Medicine I, Division of Hematology and Hemostaseology, Medical University of Vienna, Vienna, Austria; 8 Cancer Science Institute of Singapore, Singapore, Singapore; 9 Division of BioMedical Sciences, Faculty of Medicine, Memorial University of Newfoundland, St. John’s, NL Canada; 10 Department of Medicine, Northwestern University, Chicago, IL USA; 11 Institute for Research in Immunology and Cancer (IRIC), Department of Pharmacology and Physiology, Université de Montréal, Montréal, QC H3C 3J7 Canada; 12 Present Address: MOMA Therapeutics, Cambridge, MA USA

**Keywords:** Transcriptional regulatory elements, Myelopoiesis, Transcription factors, Haematopoietic stem cells

## Abstract

The Ets transcription factor PU.1 is essential for inducing the differentiation of monocytes, macrophages, and B cells in fetal liver and adult bone marrow. PU.1 controls hematopoietic differentiation through physical interactions with other transcription factors, such as C/EBPα and the AP-1 family member c-Jun. We found that PU.1 recruits c-Jun to promoters without the AP-1 binding sites. To address the functional importance of this interaction, we generated PU.1 point mutants that do not bind c-Jun while maintaining normal DNA binding affinity. These mutants lost the ability to transactivate a target reporter that requires a physical PU.1-c-Jun interaction, and did not induce monocyte/macrophage differentiation of PU.1-deficient cells. Knock-in mice carrying these point mutations displayed an almost complete block in hematopoiesis and perinatal lethality. While the PU.1 mutants were expressed in hematopoietic stem and early progenitor cells, myeloid differentiation was severely blocked, leading to an almost complete loss of mature hematopoietic cells. Differentiation into mature macrophages could be restored by expressing PU.1 mutant fused to c-Jun, demonstrating that a physical PU.1-c-Jun interaction is crucial for the transactivation of PU.1 target genes required for myeloid commitment and normal PU.1 function in vivo during macrophage differentiation.

## Abstract (web summary)

The transcription factor PU.1 recruits c-Jun as a co-activator to promoters without AP-1 binding sites, and mice with point mutations in PU.1 that disrupts the interaction between PU1 and c-Jun have defects in PU.1 dependent blood lineages, including macrophages and B cells.

## Introduction

The differentiation of hematopoietic stem cells (HSC) to various mature blood cell lineages is a process tightly regulated by the concerted action of a network of transcription factors, integrating signaling cascades triggered by extrinsic stimuli. PU.1 has been identified as an essential transcriptional regulator of hematopoiesis, required at multiple stages of myeloid and lymphoid differentiation. PU.1 can be detected in HSC, at higher levels in the common myeloid progenitors (CMP), and at high expression levels in granulocyte-macrophage progenitors (GMP) and mature myelomonocytic cells^1^. Within the lymphoid lineage, it is expressed in common lymphoid progenitors (CLP) and in pro-B and mature B lymphocytes^2^. PU.1 regulates the expression of almost all myeloid genes, including the cytokine receptors M-CSF-R^3,4^, GM-CSF-R^5^, and G-CSF-R^6^.

Loss of PU.1 expression leads to perinatal death with severe defects in the lymphoid and the myeloid lineages with a lack of monocytes/macrophages, B and T cells at birth. Additionally, studies using conditional PU.1 knock-out mice suggest that PU.1 plays an important role in the maintenance of the adult HSC pool and is required for the generation of CMP and CLP. While the loss of PU.1 in CMP prevents their maturation, it does not affect the proliferation of myeloblastic cells in culture^7^. However, PU.1 restricts excessive divisions of both normal hematopoietic stem cells (HSC)^8^ and leukemia “stem cells”^9^. At the same time, reduction of PU.1 levels below 20% of wild-type levels^10^, as well as Mx1-Cre-mediated conditional deletion of PU.1 in the hematopoietic lineages, have been shown to cause leukemias in adult mice^11^.

Aside from binding to cognate DNA sequences in the promoters and enhancers of its target genes, PU.1 also binds to multiple other transcription factors. Known partners include GATA-1^12–18^, GATA-2^19^, GATA-3^19–21^, RUNX1 (AML1)^22^, C/EBPα^23^, C/EBPβ^24^, Ski^25^, Evi-1^26^, Rb^27^, Bcl6^28^, IRF4^29^, and ICSBP/IRF8^30^. These interactions can modulate the transcriptional activity of PU.1. One example of an antagonistic interaction is the one described for PU.1 and GATA-1, which has been proposed as a general model for the initiation and resolution of mixed lineage states occurring during the differentiation of multipotent progenitors^17,31,32^. It further emphasizes that direct physical interactions between transcription factors are critical events for hematopoietic cell differentiation.

An example of a co-operative activation of target genes has been described for PU.1 and c-Jun, a member of the AP-1 transcription factor family, which includes JunB, JunD, and the Fos family, all of which are expressed in hematopoietic cells^33,34^. The AP-1 family has diverse effects, including the promotion of proliferation, differentiation, and apoptosis^35,36^. In the course of hematopoietic differentiation, c-Jun is upregulated during monocytic differentiation of myeloid cell lines^33,37–39^, and expressing a dominant negative c-Jun blocks monocytic differentiation^40^. When acting as part of the AP-1 complex, c-Jun is a bona fide transcription factor and interacts with a specific DNA-binding site, either as a homodimer or as a heterodimer with other AP-1 family members. However, c-Jun can play a different role when it interacts with PU.1. We previously reported that c-Jun directly binds and coactivates PU.1 on the M-CSF-R and IL-1β promoters, which lack AP-1 binding sites, demonstrating that c-Jun acts in these cases as a coactivator of PU.1 function, rather than as a classic transcription factor binding DNA^41,42^. The PU.1 region contacting c-Jun has been mapped to the Ets domain, whose three-dimensional structure has been extensively characterized^43,44^. This winged helix-turn-helix motif is conserved among Ets family members and serves both as a DNA-binding domain and a protein interaction domain with c-Jun^45^.

To address the importance of the interaction of PU.1 and c-Jun for hematopoietic cell differentiation, we generated and identified PU.1 mutants that do not physically interact with c-Jun but still retain intact DNA-binding capabilities. We isolated three mutants that satisfied these two criteria. In contrast to wild-type PU.1, these mutants exhibited impaired transcriptional activation ability and were unable to induce monocytic differentiation of a PU.1-deficient cell line. Moreover, mice carrying these mutations displayed an early block in myeloid differentiation and died perinatally. These results suggest that the induction of myeloid cell differentiation by PU.1 is dependent on the interaction with the PU.1 coactivator c-Jun. These findings are in line with the proposed mechanism of AML1-ETO-mediated myeloid leukemias in which the AML1-ETO fusion disrupts PU.1-c-Jun interaction and thereby leads to a differentiation block and malignant transformation^46^.

## Results

### Generation of PU.1 mutants defective in c-Jun interaction

**Fig. 1:**
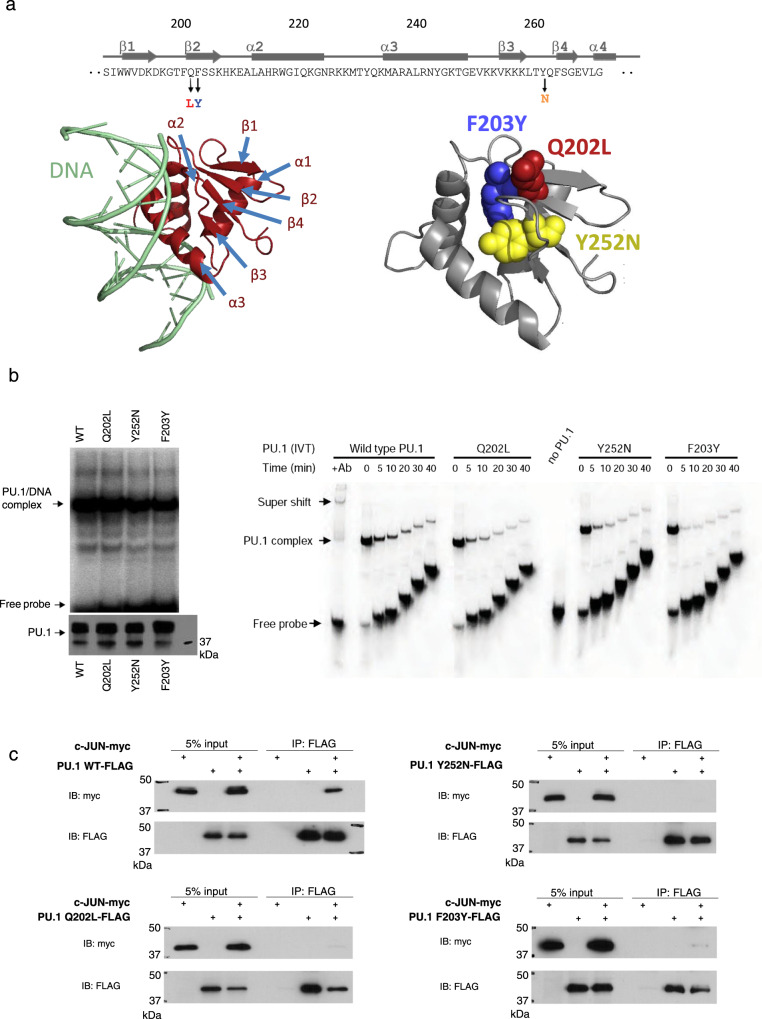
Identification and analysis of PU.1 interaction mutants that retain DNA binding. **a** Identified mutations in the beta-sheets of the human PU.1 Ets domain. The primary protein sequence, the position of the mutations, and their location within the 3D structure of the crystal of the Ets domain bound to DNA are shown. Crystal structure used PDB:1PUE. **b** The PU.1 mutants retain DNA-binding ability similar to that of wild-type PU.1(WT). Left panel: EMSA assay using a radiolabeled DNA probe containing the PU.1 binding site of the M-CSFR promoter. Equal amounts of WT and mutant PU.1 proteins were used for EMSA, as shown on the western blot probed with PU.1 antibody (below). Right panel: Following incubation of in vitro-translated (IVT) PU.1 proteins with labeled DNA, equal amounts of samples reflecting increasing times of competition were subsequently loaded onto the gel at the indicated time intervals. Probe alone (“no PU.1”) and a sample in which the PU.1-DNA complex was super-shifted with anti-PU.1 antibody (“+Ab”) were added as controls. Assays were repeated three times with representative blots shown. **c** Co-immunoprecipitation assays demonstrate the lack of interaction between mutant PU.1 proteins and c-Jun. Immunoprecipitation (IP) was carried out with anti-Flag antibody directed against Flag-tagged PU.1 and the immunoprecipitate blotted (IB) with anti-myc antibody recognizing myc-tagged c-Jun (lower panel). Assays were repeated three times with a representative blot shown. Source data for the blots can be found in Supplemental Data 1.

To address the functional importance of the PU.1-c-Jun interaction for target gene activation, we performed a mutagenesis screen to identify PU.1 point mutations that specifically abolish binding to c-Jun while maintaining PU.1-DNA-binding. Briefly, we generated a library of PU.1 mutants by random PCR mutagenesis^47^ and tested these mutants in the yeast split-hybrid system (Supplementary Methods) for c-Jun interaction as previously described^48^. More information on the screen is shown in Supplementary Fig. S1. Mutants defective in c-Jun binding were analyzed by electrophoretic mobility shift assay (EMSA) for their DNA-binding capability. We identified three mutations that stringently met these two criteria (Fig. 1a). The Y252N mutation identified in this screen is located in the β4 beta-sheet of the Ets domain. This is in accordance with previous studies that mapped PU.1-c-Jun interactions to the β3/β4 region, comprising amino acids 243–254 of PU.1^41^. In addition, we found two adjacent c-Jun binding-deficient mutations (Q202L and F203Y) located in the β2 region of PU.1^44,49^. This region has been previously implicated in protein–protein interactions, particularly between PU.1 and C/EBPβ proteins^24^. Importantly, the crystal structure of the PU.1-DNA-binding domain bound to DNA demonstrates that the β2 and β3/β4 regions are directly adjacent and that the mutated amino acids are not located on the DNA-binding surface of the Ets domain^44,49,50^ (Fig. 1a).

To determine more precisely their DNA-binding affinities, we performed a dissociation gel shift assay, in which each PU.1 protein was first incubated with a labeled PU.1 binding site oligonucleotide, and subsequently competed with an excess of unlabeled competitor oligonucleotide to measure the dissociation of the labeled complex over time. As shown in Fig. 1b, the Q202L and Y252N mutants bound to DNA with an affinity comparable to that of wild-type, while the F203Y mutant showed slightly lower affinity. As a control, none of the PU.1 proteins bound a similar DNA probe with a disrupted PU.1 site.

To ascertain that the isolated PU.1 mutants lost the ability to interact with full-length c-Jun in mammalian cells, we co-expressed the PU.1 mutants and c-Jun and performed co-immunoprecipitation experiments (Supplementary Methods). As shown in Fig. 1c, all three PU.1 mutants described here lost the c-Jun interaction in 293 T cells. Therefore, we have identified PU.1 mutations that meet the criteria of conserved DNA-binding and disrupted c-Jun interaction.

### PU.1 mutants have an impaired in vitro transactivation and differentiation potential

**Fig. 2:**
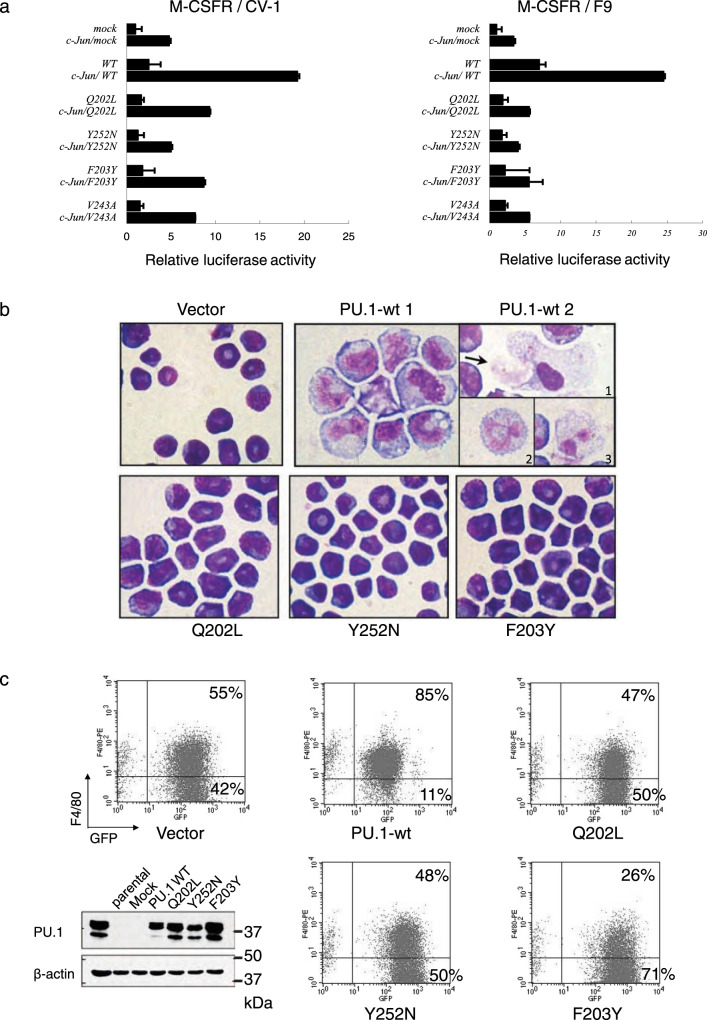
PU.1 mutants show impaired differentiation potential. **a** PU.1 mutants have impaired transactivation potential. F9 cells (no endogenous c-Jun) and CV-1 cells (expressing c-Jun) were transfected with a PU.1-responsive human M-CSFR promoter-luciferase reporter and either PU.1 alone or in combination with c-Jun to test coactivation potential. Mean of values of three independent experiments (*n* = 3) ±SD are shown. **b** PU.1 mutants fail to induce macrophage differentiation in a PU.1-deficient progenitor cell line. Wright-Giemsa staining of wild-type PU.1 (PU.1-wt) and mutant PU.1 (Q202L, Y252N, and F203Y) transduced 503 cells at day 14 cultured in differentiation medium. Magnification was ×1000. In contrast to only vector-transduced cells (Vector), 503 cells transduced with wild-type PU.1 differentiate into monocytes. The arrow (PU.1-wt 2-1) points to a cell being phagocytosed by a macrophage, as well as mature granulocytes with segmented nuclei (PU.1-wt 2–2 and 2–3). 503 cells transduced with the PU.1 mutants showed immature myeloid blasts similar to mock-transduced or parental 503 cells. Three independent experiments were performed with representative images shown. **c** Cell surface expression of the late macrophage marker F4/80 correlates with morphological signs of differentiation. 503 cells were transduced with wild-type (PU.1-wt) or mutant PU.1(Q202L, Y252N and F203Y), or with empty retroviral expression vector (Vector). Infected (EGFP^+^) cells were sorted, cultured for 10 days in differentiation medium and analyzed by flow cytometry for expression of F4/80 and EGFP expression. Protein extracts of the sorted cells were used for western blots analysis to assess PU.1 expression levels. (Fig. 2c, lower left panel). Three independent experiments were performed with representative images shown. Source data for the blot can be found in Supplemental Data 1.

To assess the impact of the impaired PU.1-c-Jun interaction on PU.1 transcriptional activity, we performed luciferase assays in either CV-1 cells, which contain endogenous c-Jun, or F9 cells that lack it. Cells were co-transfected with the previously described PU.1-responsive human M-CSFR promoter reporter^41^ and PU.1 wild-type or mutant (Q202L, F203Y, and Y252N) expression constructs. Additionally, c-Jun was co-expressed to test its coactivation potential. Similar to what we have shown before, wild-type PU.1 alone activated the M-CSFR promoter (3-fold in CV-1 cells, 7-fold in F9 cells). Co-transfection of PU.1 and c-Jun resulted in a synergistic activation (15-fold in CV-1 cells, 25-fold in F9 cells) (Fig. 2a). In the F9 cells, The PU.1 mutants were able to activate reporter transcription only 2-fold (compared to 7-fold for wild-type) in the absence of c-Jun, and only 3.5-fold for Y252N and 5-fold for Q202L and F203Y in the presence of c-Jun protein (compared to 25-fold for wild-type PU.1). Hence, the PU.1 mutants failed to synergize with c-Jun to activate transcription of the M-CSFR promoter, suggesting that the PU.1-c-Jun interaction is required for robust transcriptional activation of this target gene. Similar results were obtained with a minimal promoter containing four PU.1 binding sites, demonstrating that the synergistic activation by PU.1 and c-Jun is dependent solely upon PU.1 sites and, furthermore, is not restricted to the M-CSFR promoter. To exclude the possibility that the mutations affect the predominantly nuclear localization of the PU.1 protein, we studied the subcellular localization and observed a similar localization pattern of the wild-type and mutant proteins (Supplementary Fig. S2). Taken together, these results suggest that a direct interaction between PU.1 and c-Jun is necessary for robust activation of certain PU.1 target genes.

Given the evidence of a crucial function of c-Jun in monocytic differentiation^51–53^, we tested the implications of the disrupted PU.1-c-Jun interaction on monocytic differentiation. For this, we reintroduced either wild-type or mutated PU.1 into the PU.1-deficient, immature myeloid cell line 503^54^. Wild-type PU.1 and mutants (Q202L, F203Y, and Y252N) were subcloned into the retroviral vector MIGRI^55^, transduced into 503 cells, and sorted for EGFP expression. Upon 10 days of culture with M-CSF, wild-type PU.1/503 cells clearly differentiated into monocytes (featuring large, light-blue cytoplasm and cleaved nucleus), as well as into macrophages (adherent to the dish and performing phagocytosis) and mature granulocytes. In contrast, 503 cells transduced with PU.1 mutants were not adherent and had immature myeloid blast features with typical dense blue, round-shaped nuclei and small cytoplasm, similar to the mock-transduced 503 cells (Fig. 2b).

The failure of mutant PU.1 to induce monocytic differentiation was further assessed by monitoring cell surface expression of F4/80, a marker of mature monocytes and macrophages. Following sorting, surface markers for monocytic differentiation were analyzed by flow cytometry on day 7. While the addition of wild-type PU.1 promoted monocytic differentiation, as marked by F4/80 expression, none of the three mutants was able to increase the number of cells expressing F4/80 compared to mock-transduced cells. Similar amounts of PU.1 protein were detected in sorted EGFP^+^ cells by Western blot analysis, demonstrating that the inability to differentiate was not caused by lower PU.1 protein concentrations (Fig. 2c). These results indicate that the PU.1 mutants have an impaired in vitro transactivation and lack the potential to induce myeloid differentiation.

### PU.1 mutant mice display an early lymphoid-myeloid differentiation block

**Fig. 3:**
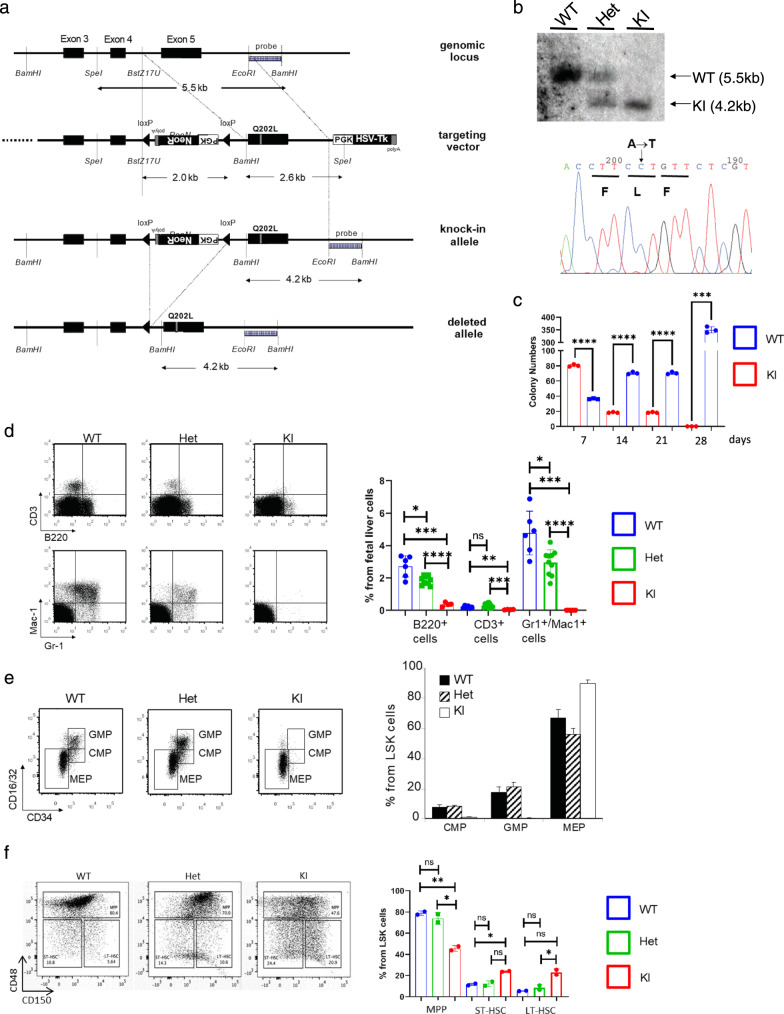
Generation of PU.1 knock-in mice and phenotypic analysis of their hematopoietic compartments. **a** Knock-in strategy. The point mutations (Q202L mutation shown here) were introduced into exon 5 by site-directed mutagenesis. Location of restriction sites within the targeted region and corresponding fragment sizes of digested DNA are shown. Cre-mediated recombination selectively excises the region between the loxP sites, comprising the neomycin selection cassette. Location of the DNA probe to screen for successful gene targeting is shown. **b** Left panel: Southern blot analysis of ES cell DNA after restriction with BamHI and SpeI shows wild-type (WT) and targeted (KI) alleles with their respective sizes, as marked in **a**. Right panel: verification of the point mutations by sequencing. The base pair change leading to the Q202L amino acid mutation was confirmed as indicated. Three independent experiments were performed with representative images shown. **c** The fetal liver cells of KI mice have self-renew ability. Total fetal liver cells of both WT and KI E14.5–16.5 mice were seed in M3434 methylcellulose containing SCF, IL-3, IL-6, and EPO. The total colony number was counted and same number of cells were replated in fresh medium every 7 days. Three independent experiments were performed. **d**–**f** Flow cytometry analysis of E14.5–16.5 fetal liver cells. The relative contribution of the listed cell populations to total live (DAPI^−^) fetal liver cells is shown as the mean of values from quadruplicates ±SD. Representative FACS profiles are shown for wild-type (WT), heterozygous (Het) and homozygous PU.1 Q202L knock-in cells (KI). Statistical p-values are control vs KI are as follows—for7 days, *p* < 0.0001; 14 days, *p* < 0.0001; 21 days, *p* < 0.0001; 28 days, *p* = 0.0004. **d** Analysis of mature B cell (B220^+^), T cell (CD3^+^), and myeloid (Gr-1^+^ Mac-1^+^) compartments. Statistical values are as follows—for B220 + cells—Het vs WT, *p* = 0.020; KI vs WT *p* = 0.002; KI vs Het, *p* < 0.0001. For CD3^+^ cells—Het vs WT, *p* = 0.058; KI vs WT *p* = 0.0026; KI vs Het, *p* = 0.002. For Gr1^+^Mac1^+^ cells—Het vs WT, *p* = 0.020; KI vs WT *p* = 0.0003; KI vs Het, *p* < 0.0001. **e** Analysis of hematopoietic lin^−^ c-kit^+^ Sca-1^−^ progenitor cell compartment. Distinct populations are marked in boxes: MEP (CD34^−^ CD16/32^−^), CMP (CD34^+^ CD16/32^low^), GMP (CD34^+^ CD16/32^hi^). **f** Analysis of hematopoietic LSK (c-kit^+^ Sca-1^+^ lin^−^) cells. Distinct populations are marked: ST-HSC (CD34^+^ Flt3^−^/CD48^−^ CD150^−^), LT-HSC (CD34^−^ Flt3^−^/ CD48^−^ CD150^+^), MPP (CD34^−^ Flt3^+^/CD48^+^). Statistical *p*-values are as follows—for MPP cells—Het vs WT, *p* = 0.41; KI vs WT *p* = 0.0067; KI vs Het, *p* = 0.049. For ST-HSC cells—Het vs WT, *p* = 0.77; KI vs WT *p* = 0.016; KI vs Het, *p* = 0.094. For LT-HSC cells—Het vs WT, *p* = 0.37; KI vs WT *p* = 0.079; KI vs Het, *p* = 0.041. All comparisons tested using two-tailed *t*-test with Welch’s Correction. ns *p* > 0.05, *p < 0.05, **p < 0.01, ***p < 0.001, ****p < 0.0001. The experiments for **d**, **f** were validated through three independent experiments. The experiment for **e** was validated through two independent experiments.

The obvious impact of the point mutations on monocytic differentiation in cell culture prompted us to study the in vivo importance of PU.1-c-Jun interactions. We generated mice with a targeted mutation of the PU.1 gene, introducing the identified single-point mutations (Fig. 3a, b). Three independent knock-in mouse lines were established, carrying the Q202L, F203Y, or Y252N mutation, respectively. In the following, we will focus on the Q202L mutation, which showed the highest DNA-binding ability in vitro (Fig. 1b), but the results obtained with the F203Y mutation closely match those reported here for the Q202L mutation.

When bred to homozygosity, the Q202L mutation knock-in (KI) mice were either born dead or died during the first day after birth. Analysis of litters at embryonic day 14.5–16.5 revealed that homozygous pups developed with normal Mendelian ratios. As the knock-in mice did not survive to the adult stage, we evaluated the progenitor populations in the fetal liver. To examine the role of the PU.1-c-Jun interaction on HSC self-renewal, we employed serial replating of total fetal liver cells from the WT or Q202L PU.1 mutant. Strikingly, abolishing the PU.1-c-Jun interaction impaired the ability of fetal liver cells to proliferate (Fig. 3c).

Further characterization of the fetal liver of these embryos revealed the complete absence of mature granulocytic and monocytic cells. In addition, there was a strong reduction in the total numbers B and T lymphocytes (Fig. 3d).

Next, we characterized the fetal liver progenitor compartment. The analysis of the fetal liver progenitor compartment showed a substantial reduction of common CMP and GMP, but not of myeloid-erythroid progenitors (MEP), in which PU.1 is not essential (Fig. 3e). To characterize the HSC compartment by staining for CD48 and CD150, abolishing the PU.1 c-Jun interaction caused a statistically significant decrease in the number of the multipotent progenitor (MPP) population with a concomitant accumulation of both long-term HSCs (LT-HSC) and short-term HSCs (ST-HSCs) (Fig. 3f). These results further support a differentiation block downstream of the HSC stage in the absence of a functional PU.1-c-Jun interaction.

Protein expression analysis using whole cell extracts of the unfractionated fetal liver did not show any detectable PU.1 expression in the mutants. To test whether this was caused by the lack of the cells normally expressing PU.1 (B cells, granulocytes, monocytes), or by a failure of the targeted construct to generate mRNA and protein, sorted hematopoietic stem and progenitor cells were analyzed for PU.1 transcription by quantitative real-time PCR (qPCR) (Supplementary Fig. S3). The c-kit^+^ Sca-1^−^ lin^−^ progenitor cells that express high levels of CD16/CD32 (FcγRII/III, similar to GMP), but low levels of CD34, have high levels of PU.1 transcript, similar to wild-type GMP. We sorted this population and performed western blot analysis of the PU.1 protein using comparable numbers of wild-type GMP and this aberrant population found in the mutant mice. Mutant PU.1 protein was indeed expressed (Supplementary Fig. S3), suggesting that the loss of the hematopoietic cells downstream of these cells is caused by a functionally defective PU.1 protein rather than by reduced PU.1 levels. This is in contrast to other PU.1 models, such as the previously characterized PU.1 URE knock-out model or the PU.1 conditional knock-out model^7,10,11^.

To ensure that the point mutation specifically abolishes the PU.1-c-Jun interaction, we tested the capability of PU.1 to interact with other transcription factors. As shown in Supplementary Fig. S4, the interactions of mutant PU.1 with C/EBPα, as well as with GATA-2 remain intact, albeit slightly decreased. These results strongly suggest that physical PU.1-c-Jun interaction is crucial for early lympho-myeloid differentiation in vivo.

### Expression of a mutant PU.1 fused to c-Jun partially rescues the differentiation block

**Fig. 4:**
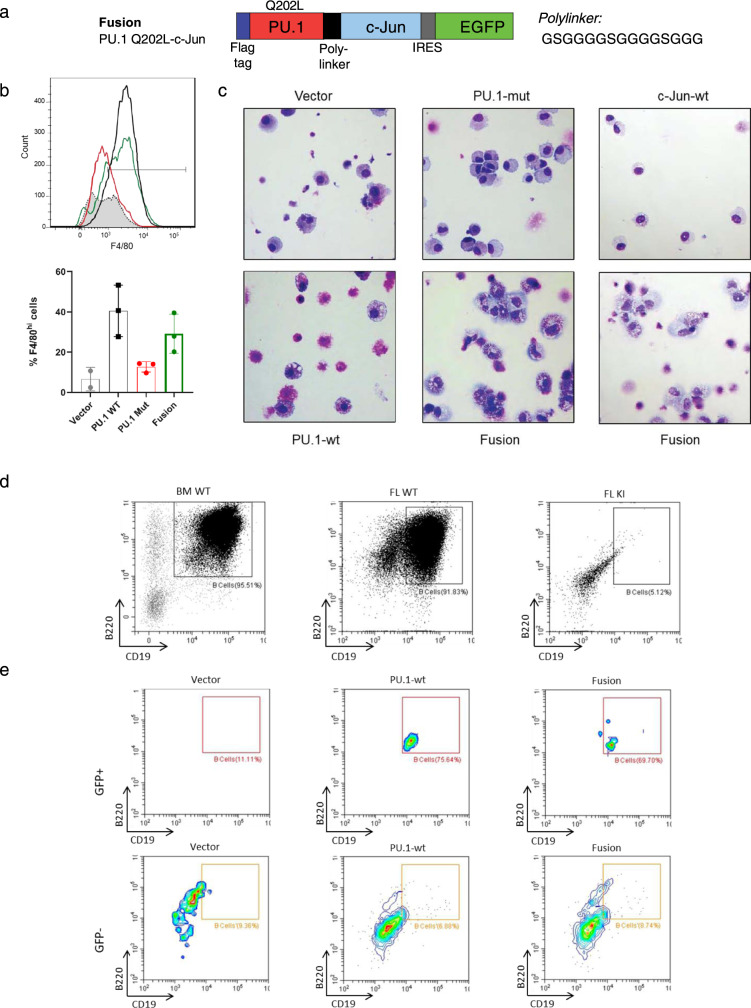
Fusion of mutant PU.1 with wild-type c-Jun rescues macrophage differentiation and B-cell differentiation. **a** Generation of a PU.1 Q202L-c-Jun fusion protein. The rescue construct consists of the PU.1 mutant Q202L with an N-terminal Flag tag fused in-frame via a flexible polylinker with the N-terminus of c-Jun. The construct was cloned into the retroviral MIGR1 vector containing IRES-GFP for selection. **b** Differentiation in cell culture of sorted KI fetal liver LSK cells transduced with MIGR1 retroviruses expressing GFP alone (Vector), GFP and wild-type PU.1 (PU.1-wt), GFP and Q202L mutant PU.1 (PU.1-mut), or GFP and Q202L mutant PU.1 fused with wild-type c-Jun (Fusion). After 14 days culture in M3434 methylcellulose containing SCF, IL-3, IL-6, and EPO, the percentage of F4/80^+^ mature macrophages within live GFP^+^ Gr-1^-^ cells was measured for three independent experiments. Bar graphs quantifying the increase in the F4/80^hi^ mature macrophages is shown on the right. Results were validated through two independent experiments. **c** Morphology of the cultured cells after 14 days in culture. Staining and imaging was performed once. **d**, **e** Differentiation in cell culture of sorted wild-type bone marrow (BM WT), wild-type fetal liver (FL WT), and mutant fetal liver (FL KI) LSK cells. Three independent experiments were performed with representative results shown (**d**), and FL KI LSK cells transduced with Vector, PU.1-wt and Fusion MIGR1 retroviruses (**e**). After 14 days culture in OP9 stromal cell containing Flt3-L and IL-7, the percentage of B220^+^/CD19^+^ mature B cells within live GFP^+^/GFP^−^ CD45^+^ CD3^−^ cells was measured. Bar graphs quantifying the percentage of the B cells are shown in Supplementary Fig. S6.

To demonstrate that the reduced transcriptional activity of PU.1 mutants was caused specifically by the failure to interact with and recruit c-Jun to target promoters, we performed rescue experiments using mutant PU.1 fused to c-Jun via a flexible poly^-^linker (Fig. 4a). Newborn liver cells were isolated and transduced with retroviruses carrying different expression constructs, as well as enhanced green fluorescent protein (EGFP) driven by an internal ribosomal entry sequence (IRES). Transduced EGFP^+^ cells were subsequently isolated by flow cytometry and cultured for 14 days in a semi-solid M3434 medium containing SCF, IL-3, IL-6, and EPO. Expression of mutant PU.1 alone was not sufficient to overcome the differentiation block caused by the absence of wild-type PU.1 at an early monomyelocytic stage, observed in KI fetal liver. The cells kept an immature morphology similar to the mock-transduced cells. However, the introduction of either wild-type PU.1, or mutant PU.1 fused to c-Jun triggered differentiation into mature macrophages with large, highly vacuolated cytoplasm and small eccentric nucleus expressing high levels of the late monocyte marker F4/80 (Fig. 4b, c). Expression of c-Jun alone did not result in an upregulation of monocytic differentiation and had a negative impact on colony formation (Fig. 4c). These results demonstrate that the physical PU.1-c-Jun interaction is crucial for early myeloid differentiation in vivo. Furthermore, expression of the PU.1-c-Jun fusion protein rescued B-cell numbers (Fig. 4d, e).

### Loss of interaction of PU.1 with c-Jun affects a subset of PU.1 target genes

**Fig. 5:**
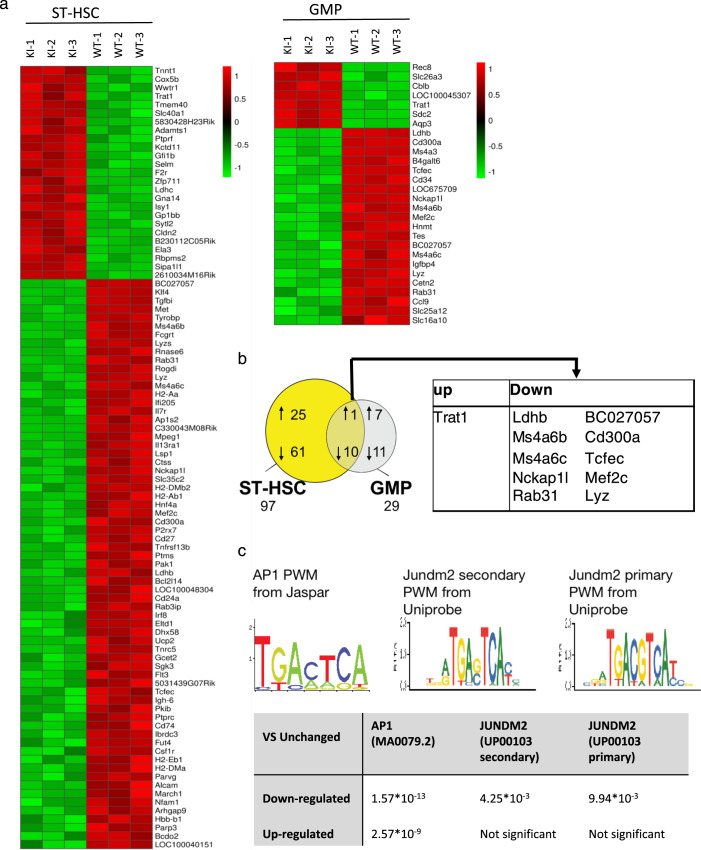
Loss of interaction with c-Jun affects a subset of PU.1 target genes characterized by PU.1 sites lacking adjacent AP-1 site. **a** Genome-wide mRNA expression profiles of ST-HSC (CD150^−^ CD48^−^ LSK, left) and GMP (lin^−^ ckit^+^Sca1^−^ CD16/32^hi^ CD34^+^, right) were generated using Affymetrix MG430v2 microarrays. Hierarchical clustering of genes differentially expressed in PU.1 KI cells. Only genes with a log2 fold change >2.0 were considered differentially expressed. **b** Venn diagram showing the overlap of differentially expressed features between wild-type and mutant samples. Numbers below the populations correspond to the sum of the up- and downregulated genes in the mutant population compared to the wild-type counterpart. The genes differentially expressed in both populations are listed in the table below the diagram. **c** AP-1 consensus motifs found in unaffected gene promoters. Sequence logos that correspond to enriched sequence elements, as identified by de novo motif analysis in PU.1 ChIP-seq regions in genes with unchanged expression in the PU.1 mutant samples are shown. The table lists the statistical significance of the occurrence of the motifs. Consensus AP-1 motifs as well as a closely related motif with an additional base at the core of the motif corresponding to the Jundm2 consensus are shown.

To identify PU.1 target genes that help explain the severe differentiation block observed in the KI mice, and to assess the fraction of PU.1 target genes that are affected by the mutation, we sorted ST-HSC (CD150^−^ CD48^−^ LSK) from the fetal liver of KI and wild-type embryos (WT), a differentiation stage equally present in wild-type and mutant mice, as well as wild-type GMP (lin^−^ c-kit^+^ Sca-1^−^ CD16/32^hi^ CD34^+^) and the GMP-like population (lin^−^ c-kit^+^ Sca-1^−^ CD16/32^hi^ CD34^int^) appearing in mutant mice. We purified the RNA, performed cDNA synthesis, linear amplification, and microarray analysis to compare global gene expression changes between the samples. Significant differences in gene expression patterns between wild-type and mutant samples occurred already at the ST-HSC stage. Using the SAM algorithm to assess significant changes between the populations, we identified 25 genes that were upregulated and 61 genes that were downregulated in the mutant compared to wild-type ST-HSC, showing that mutation of PU.1 already affects these very immature hematopoietic precursor cells (Fig. 5a, b). RT-qPCR was also utilized to further validate the expression differences observed (Supplementary Fig. S5, Supplemental Table 1) To assess the importance of the PU.1-c-Jun interactions, we focused on the gene expression of direct PU.1 target genes that have been experimentally determined previously^56^. We observed downregulation of a number of known targets in mutant ST-HSC and GMP-like cells, including, as expected, the gene encoding the M-CSF-R, but also other genes involved in macrophage recruitment and differentiation, such as the cysteine protease Cathepsin S (Ctss), and Tyrobp (DAP-12), an adapter protein coupling M-CSF-R signaling to stabilization and nuclear translocation of β-catenin^57^. Similarly, the IL-7 receptor alpha chain (Il7r), important for B-lineage differentiation, was significantly downregulated already in the ST-HSC population. In contrast, other direct PU.1 target genes, such as Blnk, the LPS co-receptor CD14, as well as Ly96, an adapter of TLR4 involved in LPS responsiveness, were unaffected by the mutation (Tables 1,2). Of note, neither PU.1, nor c-Jun mRNA expression were significantly altered in the sorted cell fractions from the PU.1 mutant mice (Table 3).

**Table 1:** Selected genes dysregulated in PU.1 mutant ST-HSC.

Gene symbol	Gene title	Probe ID	Fold change (log_2_)
	**MHC-associated genes**		
*H2-Aa*	Histocompatibility 2, class II antigen A, alpha	1435290_x_at	−8.56
*Cd74*	CD74 antigen (invariant polypeptide of major histocompatibility complex, class II antigen-associated)	1425519_a_at	−7.03
*H2-Eb1*	Histocompatibility 2, class II antigen E beta	1417025_at	−6.30
*H2-Ab1*	Histocompatibility 2, class II antigen A, beta-1	1450648_s_at	−6.28
*H2-DMb2*	Histocompatibility 2, class II, locus Mb2	1443686_at	−4.71
*March1*	Membrane-associated ring finger (C3HC4) 1	1440209_at	−4.34
*H2-DMa*	Histocompatibility 2, class II, locus DMa	1422527_at	−3.42
*Fcgrt*	Fc receptor, IgG, alpha chain transporter	1416978_at	−2.67
	**Macrophage genes**		
*Ctss*	Cathepsin S	1448591_at	−7.88
*Mpeg1*	Macrophage expressed gene 1	1427076_at	−7.73
*Csf1r*	Colony stimulating factor 1 receptor	1419872_at	−6.84
*Irf8*	Interferon regulatory factor 8	1416714_at	−6.20
*Tcfec*	Transcription factor EC	1419537_at	−3.81
*Lyz*	Lysozyme	1436996_x_at	−3.25
*Lyzs*	Lysozyme	1423547_at	−2.92
	**B-cell gene**		
*Il7r*	Interleukin 7 receptor	1448575_at	−4.72
*Igh-6*	Immunoglobulin heavy chain 6 (heavy chain of IgM)	1427351_s_at	−3.16
*Gcet2*	Germinal center expressed transcript 2	1420544_at	−2.73
*Ptprc*	Protein tyrosine phosphatase, receptor type C	1422124_a_at	−2.62
*Tnfrsf13b*	Tumor necrosis factor receptor superfamily, member 13b	1423182_at	−2.57
	**T-cell genes**		
*Cd27*	CD antigen 27	1452389_at	−2.46
*Il13ra1*	Interleukin 13 receptor, alpha 1	1427165_at	−3.94

**Table 2:** All genes significantly dysregulated in PU.1 mutant GMP-like cells.

Gene symbol	Gene title	Probe ID	Fold change (log_2_)
*Ldhb*	Lactate dehydrogenase B	1434499_a_at	−7.43
*Ms4a3*	Membrane-spanning 4-domains, subfamily A, member 3	1420572_at	−6.79
*Ms4a6b*	Membrane-spanning 4-domains, subfamily A, member 6B	1418826_at	−6.06
*B4galt6*	UDP-Gal:betaGlcNAc beta-1,4- galactosyltransferase, polypeptide 6	1423228_at	−5.91
*LOC675709*	Similar to Beta-1,4-galactosyltransferase 6 (Beta-1,4-GalTase 6) (Beta4Gal-T6)	1460329_at	−5.82
*Ms4a6c*	Membrane-spanning 4-domains, subfamily A, member 6 C	1450234_at	−5.64
*Nckap1l*	NCK associated protein 1 like	1428786_at	5.09
*BC027057*	cDNA sequence BC027057	1444546_at	−5.07
*Cd300a*	CD300A antigen	1435903_at	−4.95
*Tcfec*	Transcription factor EC	1419537_at	−4.91
*LOC675709*	Similar to Beta-1,4-galactosyltransferase 6 (Beta-1,4-GalTase 6) (Beta4Gal-T6)	1435758_at	−4.40
*Mef2c*	Myocyte enhancer factor 2 C	1421027_a_at	−4.28
*Hnmt*	Histamine N-methyltransferase	1417702_a_at	−3.66
*Slc25a12*	Solute carrier family 25 (mitochondrial carrier, Aralar), member 12	1428440_at	−3.19
*Ccl9*	Chemokine (C-C motif) ligand 9	1417936_at	−3.06
*Rab31*	RAB31, member RAS oncogene family	1416165_at	−3.00
*Tes*	Testis derived transcript	1460378_a_at	−2.91
*Cd34*	CD34 antigen	1416072_at	2.87
*Slc16a10*	Solute carrier family 16 (monocarboxylic acid transporters), member 10	1436368_at	−2.67
*Lyz*	Lysozyme	1439426_x_at	−2.03
*Igfbp4*	Insulin-like growth factor binding protein 4	1423756_s_at	−1.59
*Cetn2*	Centrin 2	1418579_at	−1.10
*Cblb*	Casitas B-lineage lymphoma b	1458469_at	1.67
*Rec8*	REC8 homolog (yeast)	1419147_at	2.05
*LOC1000453*	Similar to Synaptotagmin-like	1425787_a_at	2.71
*07///Sytl3*	3///synaptotagmin-like 3		
*Sdc2*	Syndecan 2	1448545_at	2.77
*NA*	NA	1445627_at	3.29
*Trat1*	T-cell receptor associated transmembrane adaptor 1	1427532_at	3.83
*Slc26a3*	Solute carrier family 26, member 3	1421445_at	4.11
*Aqp3*	Aquaporin 3	1422008_a_at	6.35

**Table 3:** Unaffected PU.1 Target Genes.

Gene symbol	Gene title	Probe ID	Fold change (log_2_)
*Sfpi1*	SFFV proviral integration 1	1418747_at	0.12
*Jun*	Jun oncogene	1448694_at	1.01
*JunB*	JunB oncogene	1415899_at	0.13
*Blnk*	B-cell linker	1451780_at	0.08
*CD14*	CD14 antigen	1417268_at	−0.90
*Ly96*	Lymphocyte antigen 96	1449874_at	1.08

### The AP-1 motif is highly enriched in promoters of unaffected PU.1 target genes

PU.1 binding sites have been mapped by chromatin immunoprecipitation-coupled deep sequencing (ChIP-seq) in the PU.1-inducible myeloid progenitor cell line PUER^19^, as well as in peritoneal macrophages and in B cells^56,58^. To correlate the gene expression observed in our PU.1 mutant cells with putative PU.1 binding, we analyzed the published data from Heinz and colleagues^58^, obtained with PUER cells. First, we divided all expressed genes into three groups based on their differential expression within the PU.1 mutant populations: unaffected, downregulated or upregulated in comparison with wild-type cells. We then considered the PU.1 binding sites localized within each gene or its ±50 kb flanking region as potential PU.1 target sites. We subsequently retrieved the corresponding DNA sequences and performed a Position Weight Matrix (PWM) search using PSCAN^59^ on current public transcription factor databases: Jaspar^60^ and Uniprobe^61^. The PWM search allowed us to identify transcription factor binding sites associated with PU.1 within the three groups. Jaspar uses the consensus sequence TGANTCA as AP-1 matrix (Fig. 5c, left panel), while Uniprobe uses two matrices describing the binding site of the AP-1 transcription factors: The first matrix corresponds to the canonical AP-1 matrix, while the second has an additional nonconserved residue in the center of the motif (TGANNTCA) (Fig. 5c, middle and right panel) and is the consensus site for Jundm2, another member of the Jun family.

According to PScan, when searching the promoter region (−950 ± 50 bp) of the unaffected genes noted in Table 3 against the TRANSFAC database, the top transcription factor candidate was AP-1 with *p* = 0.0012. The 2nd and 3rd candidates are STAT3 (p = 0.0025) and BACH2 (*p* = 0.0041). It is therefore evident that the best matching TF motif identified in the unaffected genes is AP-1. We also noted the absence of Pu.1 partner TF’s such as CEBPA (*p* = 0.26) and GATA-2 (*p* = 0.32), GATA-1 (*p* = 0.29), MYB (*p* = 0.42), and AML1 (*p* = 0.51).

As shown in Fig. 5c, we found a noticeable enrichment of the AP-1-binding motif in the regions immunoprecipitated by PU.1 in genes that were not affected by the PU.1 mutation. Moreover, the Jundm2 primary matrix, which allows a gap of 2 nucleotides between both halves of the core motif, is significantly over-represented in the unchanged group compared to the downregulated group. This finding could explain how a subset of bona fide PU.1 target genes is still activated by the PU.1 mutant that cannot directly recruit c-Jun to form a transcriptional coactivator complex. Therefore, the direct interaction of PU.1 and c-Jun appears to be crucial in regulatory regions without AP-1 sites adjacent to PU.1, while the binding of both factors in close proximity to each other might alleviate the requirement of a robust direct interaction to form a ternary complex.

## Discussion

PU.1 is a master regulator of myelopoiesis and lymphopoiesis due to its crucial role in directing the generation of monocytes and lymphocytes^62,63^. Furthermore, PU.1 cooperates with several transcription factors to orchestrate entire transcriptional networks that establish cellular identity or drive the differentiation of multipotent progenitor cells into distinct lineages^56,58,64^. We have previously identified c-Jun to be a direct interaction partner of PU.1 that plays a role in monocytic differentiation^41^. PU.1 interacts via the Ets domain with the basic domain of c-Jun in vitro^45^, and the recruitment of c-Jun by PU.1 enhances the activity of the M-CSF-R promoter, which lacks an AP-1-DNA-binding site^41^. We investigated the importance of this interaction for PU.1 function using PU.1 mutants which fail to interact with c-Jun but still retained DNA-binding ability. These mutations are located in the β2 and β3/β4 regions of the Ets domain, on a molecular surface facing away from the DNA, suggesting that this surface area participates directly in PU.1-c-Jun protein–protein interactions. We did not identify any mutations in the PEST domain, suggesting that this domain, which mediates interactions with IRF factors, does not play a role in the PU.1-c-Jun interaction. Our findings are consistent with previous reports of interactions between the PU.1 Ets domain and c-Jun^41,45^. Furthermore, the PU.1 mutants maintained intact binding to C/EBPα and GATA-2, confirming the specificity of the mutation sites.

We previously showed in a PU.1 knockdown model that c-Jun overexpression can help overcome the myelomonocytic differentiation block caused by substantially reduced PU.1 expression in vitro^65^. While that model demonstrated the importance of both PU.1 and c-Jun for monocytic differentiation, it neither dissected the requirement of their interaction on the protein level nor did it address terminal macrophage differentiation. Here we provide evidence that direct interaction of PU.1 with c-Jun is absolutely required to allow PU.1-mediated macrophage differentiation.

In the present manuscript, the importance of the PU.1-c-Jun interaction was further demonstrated in vivo by the generation of a PU.1 mutant knock-in mouse model. We observed an early block of hematopoietic differentiation with an accumulation of cells at a GMP-like stage with consecutive loss of the more mature myeloid populations, which is absent in PU.1-deficient mice^7^. This suggests that the mutant PU.1 maintains residual function and might affect a subset of PU.1 target genes, presumably those genes that require recruitment of c-Jun for activation. In contrast to PU.1 knockdown and knock-out models, the observed effect is not caused by reduced PU.1 levels, as we can detect both mRNA and protein in amounts that surpass 50% of wild-type levels in the stem and progenitor cell populations (Supplementary Fig. S3). PU.1^KI/+^ mice, however, have a normal phenotype, in line with the observation that only mice expressing 20% or less of PU.1 wild-type levels show a phenotype^10^. To show that specifically the interaction of PU.1 with c-Jun is required for myeloid differentiation, we generated a rescue construct consisting of c-Jun tethered to mutant PU.1 protein and expressed it in mutant LSK cells. The in vitro data presented here show that PU.1-c-Jun interactions are essential for the differentiation of progenitors into mature monocytes and macrophages and that a fusion construct of c-Jun tethered to mutant PU.1 was able to rescue this process, while overexpression of c-Jun in the background of the PU.1 mutant did not have a beneficial effect on monocytic differentiation.

While the B and T lymphoid, as well as the myeloid lineages, were virtually absent in the fetal liver of mutant mice, MEP and erythroid cells were essentially normal, suggesting that PU.1-c-Jun interactions are not required in this lineage. Our mouse model suggests, similar to the model of Jacobsen and colleagues, that MEP are not necessarily derived from LMPP, but can branch off directly from an earlier progenitor or HSCs^66^. Our PU.1 mutant knock-in mouse model could be a useful tool to study this hypothesis.

To gain further insight into the pathways affected by the mutation, we sorted ST-HSC and the GMP-like population from mutant fetal liver and compared their gene expression profile with wild-type ST-HSC and GMP populations. We observed that a number of genes were deregulated in ST-HSC, emphasizing the fact that functional PU.1 is required for normal gene expression already at this early differentiation stage. Interestingly, closer inspection of known direct PU.1 target genes, previously characterized in immature myeloid cells, revealed a subset that was unaffected by the PU.1 point mutation, including Blnk, Ly96, and CD14. In addition, mRNA expression of PU.1 itself, but also of c-Jun and JunB, was unchanged in both ST-HSC and GMP-like cell populations.

Other groups have carried out global studies of PU.1 binding and target gene expression in both myeloid and lymphoid cells^56,58^. These studies identified a large number of PU.1 binding regions within the regulatory regions of genes that were activated upon induction of PU.1 expression, thereby being bona fide direct PU.1 target genes. Using these regions as references, we queried the list of genes expressed in the ST-HSC of the mutant mice for common binding motifs. We identified an enrichment of AP-1 sites in the regulatory regions detected by ChIP-seq in genes that were not affected by the PU.1 mutation. Additionally, a motif associated with the related AP-1 transcription factor Jdm2, consisting of a very similar binding matrix, was highly enriched in the genes that were unaffected by the PU.1 mutation compared with genes that were downregulated. These findings suggest that, in this case, direct DNA binding of c-Jun adjacent to PU.1 could allow the assembly of PU.1-c-Jun complexes even when protein–protein interactions are disrupted. In scenarios in which c-Jun binding cannot occur adjacent to PU.1, such as in the M-CSFR promoter, direct protein–protein interactions might be absolutely required for PU.1-c-Jun coactivation to occur, as our experimental data suggests. The occurrence of nonconsensus AP-1 motifs that cannot bind AP-1 complexes in the absence of adjacent Ets binding sites has been observed previously (ref. ^45^ and references therein). Here, we specifically addressed the interactions with PU.1, and we observe a very severe block in hematopoietic differentiation in vivo.

The importance of the PU.1-c-Jun interactions, as observed in our mouse model, also becomes apparent in two leukemia models in which this interaction is affected. First, ectopic expression of Evi-1 in mouse bone marrow can lead to myeloid dysplasia, caused by its association with PU.1, which competes with c-Jun binding. At the same time, Evi-1 can associate with GATA-1, blocking erythropoiesis, thereby leading to anemia^26^. As a second example, the AML1-ETO fusion protein found in t(8;21) myeloid leukemia can prevent c-Jun binding to PU.1, thereby inactivating the expression of PU.1 target genes and arresting myeloid differentiation^46^.

The relevance of the PU.1-c-Jun interaction is further corroborated by the fact that abolishing the PU.1-c-Jun interaction enhanced the self-renewal capacity of fetal liver cells in serial replating experiments (Fig. 3C). This sustained self-renewal of fetal liver cells suggests that the PU.1-c-Jun interaction is important for promoting myeloid differentiation and that the absence of the PU.1-c-Jun interaction could potentially contribute to myeloid leukemia. Such enhanced self-renewal is observed in cells transduced with the oncogenic fusions HRX-ENL^67^, AML1-ETO^68^, and MOZ-TIF2^69^.

Similar to the observed combinations of PU.1 and C/EBPβ in myeloid cells, and PU.1 and E2A in B cells^58^, the PU.1-c-Jun combination studied here might act as a beacon for the assembly of cell-type and differentiation-specific promoter complexes. It would be of interest to perform similar ChIP-seq experiments using PU.1 and c-Jun in wild-type and mutant backgrounds to analyze target gene-specific sequence motifs to further dissect this critical interaction.

## Materials and methods

### Generation of PU.1 mutants which bind DNA but do not interact with c-Jun

PU.1 point mutations in the Ets domain were generated by random PCR mutagenesis^47^. Mutants were subsequently screened by a yeast split-hybrid assay as previously described^48^. Detailed information about the screen can be found in the supplement. Mutants defective in c-Jun binding were further analyzed by electrophoretic mobility shift assay (EMSA) for their DNA-binding capability.

### Electrophoretic mobility shift assays (EMSA)

In vitro recombinant human PU.1 protein was produced by Sp6 priming using the TNT kit (Promega) and ^35^S-labeled methionine (PerkinElmer Life Sciences). Two microliters of total protein from the TNT reaction was loaded on a 6% SDS-PAGE gel to confirm efficient translation and equivalent protein production of human PU.1 wild-type and mutant proteins. Oligonucleotides derived from the CD11b promoter containing a defined PU.1 binding site were annealed to form a double-stranded probe, radiolabeled with ^32^P-γ-ATP (PerkinElmer Life Sciences) by T4 polynucleotide kinase (New England Biolabs), and purified through a Sephadex G-25 column (Roche Applied Science). EMSA was performed using previously published conditions^70^. PU.1 antibody (Santa Cruz sc-352) was used for supershift.

Dissociation gel shift assays were performed to assess the stability of complexes of mutated PU.1 protein with DNA oligonucleotides from the human CD11b promoter^41^. We confirmed by western blot that an equal amount of PU.1 wild-type and mutant proteins were in vitro-translated using 1 μg of DNA in the pcDNA3 vector. These in vitro-translated proteins were then incubated with radiolabeled oligonucleotides for 20 min on ice. After retrieving the first sample (t = 0), a 25-fold excess of unlabeled oligonucleotides was added to the reaction. Aliquots taken at the indicated time points after the addition of competitor were loaded onto 6% polyacrylamide gels, run in 0.5% TBE buffer, and analyzed by exposing the dried gels on BioMax MS film (Kodak).

### Co-immunoprecipitations and western blotting analysis

PU.1 wild-type and mutants were cloned into the mammalian expression vector pcDNA3 and labeled with an in-frame N-terminal Flag tag. Similarly, human c-Jun cDNA was cloned with an N-terminal Myc tag into pcDNA3. These constructs were transiently co-transfected into 293 T cells using Lipofectamine LTX (Invitrogen). Forty-eight hours after transfection, cells were harvested, washed in PBS, and lysed in 150 mM NaCl, 50 mM Tris-HCl (pH 7.4), 1 mM EDTA, 1% Triton X-100 and protease inhibitor cocktail (Roche) by incubating on ice for 20 minutes and passed three times through a 28-gauge syringe. After centrifugation at 16,000 × *g* for 10 min at 4 °C, the lysate was collected. Then, 40 μl of anti-FLAG M2-agarose affinity gel (Sigma) was added to the lysates and incubated at 4 °C for 2 h. The affinity gels were washed three times with TBS, and the immunoprecipitated proteins were eluted by incubation with 20 ± l of 3× FLAG peptides solution (0.5 mg/ml; Sigma) on ice for 30 minutes. For western blotting analysis, extracted proteins were separated in 10% SDS-PAGE gels and transferred to Immobilon-P (Millipore). The membranes were sequentially incubated with 5% skimmed milk, primary antibodies at 1:2000 dilution, and secondary antibodies conjugated with horseradish peroxidase at 1:5000 dilution. Then, the membranes were incubated for chemiluminescence (Luminol Reagent; Santa Cruz), and proteins were detected by X-ray film exposure. The films were visualized with a PCX-101 (KONICA MINOLTA). Western blots were probed with antibodies either c-Jun (Santa Cruz, sc-1694, H-79, JUN), PU.1(Santa Cruz, sc-352, T-21, Spi-1), FLAG (Sigma, F3165, M2), or c-Myc (Novus, NBP1-97617, PL14, MYC). WB quantification was performed using ImageJ.

### Transient transfection and luciferase reporter assays

CV-1 and F9 cells were cultured in DMEM + 10% FBS. For transcriptional activation studies, full-length wild-type and mutant PU.1 in pcDNA3, pSV1-SPORT-c-Jun, and the pXP2-M-CSF receptor promoter fused upstream of a luciferase reporter^41^, or pXP2 alone, and pRL-null as a control for transfection efficiency^71^, were transiently transfected into CV-1 or F9 cells with Lipofectamine and PlusReagent (Invitrogen). After 24 h, transfected cells were lysed with 60 μl of 1× Lysis Buffer according to the manufacturer’s protocol, and 20 μl of lysate was assayed with luciferase reagent (Promega). The remaining 40 μl of lysate was loaded onto a 10% SDS-PAGE gel and processed for western blotting with anti-PU.1 antibody (Santa Cruz sc-352) to measure PU.1 protein expression. Anti-β-tubulin (Sigma T5293 clone 2-28-33) was used to ensure the even loading of protein extracts.

### Retroviral transduction of mouse cells

The PU.1-deficient, immature myeloid cell line 503^54^ were cultured in IMDM + 10% FBS, 5% WEHI conditioned medium, and 3% BHK conditioned medium as a source of IL-3 and SCF, respectively. For retroviral transduction, wild-type and mutant PU.1 constructs were subcloned into the MIGRI vector (kindly provided by Harinder Singh) in the EcoRI site^55,72^. Retrovirus was generated by transient transfection with 5 μg of MIGRI-PU.1, 5 μg of PHCMV-A-MLVenv, and 1 μg of pGagpol (kindly provided by Carol Stocking, Heinrich-Pette-Institute, Germany) into 5 × 10^6^ Phoenix amphotropic packaging cells (Orbigen) using Lipofectamine and Plus Reagent according to the manufacturer's instruction (Invitrogen). After 48 hours, the supernatant was harvested and passed through a 0.45 μm filter (MILLEX-HA, Millipore). For retroviral transduction, 1 × 503 cells were incubated with 1 ml viral supernatant and 10 μg/ml of Hexadimethrine bromide (10868-9, Aldrich Chem. Co) in fibronection-pretreated wells (10 mg/ml of Fibronectin, F-0895 SIGMA, 1146 Becton Dickinson). After spinoculation at 800 × *g* for 120 min, 1 ml of fresh medium was added to the cells. After 24 h, a second spinoculation was carried out. Forty-eight hours after the first transduction, GFP expression in 503 cells was examined by flow cytometry. PU.1 expression was determined by Western blotting with anti-PU.1 antibody at 1:1000 dilution (Santa Cruz sc-352). The membrane was stripped with 100 mM, washed with PBS, and incubated with appropriate antibodies in PBS containing 5% calf serum for 30 min on ice in a total volume of 20 μl. The cell pellet was washed three times with PBS and suspended in PBS containing 2% calf serum. Analyses were performed with FACS can and CellQuest software (Becton Dickinson) or LSRII (Becton Dickinson) and FlowJo software (Tree Star). Fluorescence-conjugated antibodies used include anti-murine CD11b (M1/70), Gr-1 (RB6-8C5), F4/80 (BM8), B220 (RA3-6B2), CD3 (145-2C11), CD34 (RAM34), CD16/32 (93), Flt3 (A2F10), Sca-1 (D7), c-kit (2B8), and Ter119 (TER-119).

Freshly isolated lineage negative (B220^−^ CD3^−^ Ter119^−^ Gr-1^−^) c-kit^+^ Sca-1^+^ (LSK) fetal liver cells of PU.1 mutant mice were sorted by FACS and infected with retroviral MIGRI vectors containing expression constructs of wild-type or mutant PU.1, c-Jun, or fusion constructs of wild-type or mutant PU.1 with c-Jun. An empty MIGRI vector was used as a negative control (“mock”). The fusion proteins were generated by fusing full-length human PU.1 via a flexible polylinker (GSGGGGSGGGGSGGGG) to murine c-Jun.

### Serial replating assay

Harvest total fetal liver cells from both wild-type and knock-in Day 14.5–16.5 embryos (both genders), seed 2 × 10^4^ in 1 ml MethoCult™ GF M3434 (STEMCELL, USA), and plate in 35 mm dish. Culture at 37 °C, 5% CO_2_, count the colony number, and replate the same number of cells in the same condition every 7 days until the control group has no more colonies.

### In vitro differentiation of mouse cells

Infected (EGFP^+^) PU.1/503 cells were sorted by fluorescence-activated cell sorting (FACS) and cultured in differentiation medium (IMDM containing 10% FBS, 10% filtered supernatant of IL-3-producing WEHI-3 cells, 10% filtered supernatant of SCF-producing BHK-KL cells, supplemented with 10 ng/ml of recombinant murine M-CSF (416-ML, R&D Systems)) for 10 to 14 days, as indicated. Approximately 10^4^ cells were centrifuged at 500 rpm for 5 min onto glass slides and stained with Wright-Giemsa.

Murine infected (EGFP^+^) LSK cells were sorted 2 days post-infection on a Becton Dickinson FACS Aria and plated in semisolid M3434 Methocult (Stem Cell Technologies) methylcellulose medium and analyzed 14 days later. Colonies were scored visually and analyzed morphologically by Wright-Giemsa-stained cytospins.

### OP9 co-culture assay

Sort LSK from both wild-type and knock-in E14.5–16.5 fetal liver cells (both genders) and wild-type adult bone marrow (both genders, mice were aged between 2-3 months old). Seed the same number of LSK into 96-well plate with 70–90% confluent OP9 cell (Gibco^TM^ αMEM nucleosides no phenol red, Thermo Fisher Scientific, USA/20% FBS). Meanwhile, transduce MIGR1 (Vector)/PU.1-wt/Q202L-c-Jun (Fusion) into the same number of knock-in LSK using RetroNectin^®^ (TaKaRa, Japan). After 72 h culture, seed all of the transduced LSK into 96-well plate with 70–90% confluent OP9 cell as well. Change medium (X-VIVO^TM^ 15, Lonza, Switzerland/2% BSA/5 ng/ml Flt3-L/1 ng/ml IL-7, PeproTech^®^, USA/Penicillin/Streptomycin) every 3 days. On day 14, after the seeding of LSK, harvest cells and stain. Identify B cell by Flow Cytometry.

### Generation of PU.1 knock-in mice

To generate mice with the point mutations in the Ets domain, the targeting strategy is outlined in Fig. 3a. We used a targeting vector carrying exons 3–5 of PU.1 in which the point mutation was introduced by site-directed mutagenesis. For the positive selection of targeted ES cells, we inserted a neomycin selection cassette driven by the PGK promoter and flanked by loxP sites between exons 4 and 5. A negative selection cassette containing HSV-thymidine kinase driven by the PGK promoter was added to the 3’ end of the targeted region. A 1.4 kb EcoRI-BamHI fragment located just 3’ of murine PU.1 exon 5 was used as a probe for Southern blot. The correct genomic insertion was tested by Southern blot analysis, and the presence of the point mutation was verified by sequencing (Fig. 3b).

Chimeric mice were derived from embryonic stem cells that were successfully targeted by homologous recombination, and several founder lines were obtained for each construct. For the Q202L mutation, two independent founder lines were kept and analyzed. Results obtained with both lines were identical.

Mouse experiments and analysis of mouse cells were performed in compliance with institutional guidelines and approved by the Beth Israel Deaconess Medical Center Institutional Care and Use Committee (protocol # 006-2008).

### Quantitative real-time PCR

RNA isolation and DNase I treatment of FACS-sorted hematopoietic cells was carried out with the RNeasy Micro kit (Qiagen) according to the manufacturer’s instructions. After reverse transcription, the resulting cDNA was PCR-amplified using a Rotor-Gene RG 6000 (Corbett). To specifically detect wild-type and mutated PU.1, TaqMan MGB probes (Invitrogen) (wild-type: 5ʹ-CCTTCCAGTTCTCGT-3ʹ; Q202L mutant: 5ʹ-CCTTCCTGTTCTCGT-3ʹ) were used in conjunction with the following PCR primers: 5ʹ-GCATCTGGTGGGTGGACAAG-3ʹ and 5ʹ-CGCCAGCGCCTCCTT-3ʹ. Primers for GAPDH (Invitrogen) were used for reference.

### Statistics and reproducibility

Student’s t-tests were performed to determine the statistical significance of the experimental results.

### RNA isolation, amplification, and array expression analysis

ST-HSC (lin^−^ c-kit^+^ Sca-1^+^ CD150^−^ CD48^−^) and GMP (lin^−^ c-kit^+^ Sca-1^−^ CD16/32^hi^ CD34^+^) were sorted by FACS and RNA was prepared by RNeasy Micro kit (Qiagen). Ten nanograms of total RNA were used for linear amplification of cDNA using the Ovation Pico RNA Amplification System (Nugen), according to the manufacturer’s instructions. Five micrograms of cRNA were biotin-labeled using the FL-Ovation cDNA Biotin Module V2 (NuGen). Following fragmentation, the labeled cRNA of each individual sample was hybridized to Affymetrix MG430 v2 microarrays (Affymetrix) and stained according to the manufacturer’s instructions. Array data have been stored in the gene expression omnibus database (www.ncbi.nlm.nih.gov/geo/; accession no.: GSE27873) according to MIAME standards. Microarray data were normalized using the RMA method with R-Bioconductor, and the differentially expressed genes were identified using the Significance Analysis for Microarrays (SAM) algorithm^73^ in TM4-MeV software^74^ with the threshold set to 0 as a median number of falsely significant genes.

### ChIP-seq analysis, peak selection, and peak annotation

To correlate gene expression with putative PU.1 binding, we analyzed the published PU.1 chromatin immunoprecipitation-coupled deep sequencing (ChIP-seq) data from GEO GSE21512^58^, obtained with the immature myeloid PUER cell line^19^. The data obtained with uninduced (t = 0 h) cells were used as a control, and the specific signals appearing after 1 h, and 6 h were combined and considered as PU.1-bound regions. We considered all ChIP regions localized within a gene or its ±50 kb flanking region as potential PU.1 target sites regulating the expression of the aforementioned gene. The identified ChIP-seq regions were divided into three groups based on the expression of the targeted gene within the PU.1 mutant populations that we performed microarray expression analysis on: unchanged, upregulated, and downregulated. The corresponding DNA sequence was retrieved, and a Position Weight Matrix (PWM-mathematical representation of a TFBS) search was performed using PSCAN^59^.

### Crystal structure visualization

For Fig. 1, PDB: 1PUE was downloaded and representations rendered in Pymol v2.5.0^75^.

### Reporting summary

Further information on research design is available in the Nature Research Reporting Summary linked to this article.

## Supplementary information

Supplemental Information

Description of Additional Supplementary Files

Supplementary Data 1

Reporting Summary

## Data Availability

All data generated or analyzed during this study are included in this published article and its supplementary information files. Numerical source data behind main figure graphs can be found in Supplementary Data 1. Unprocessed blots can be found in Supplementary Fig. S7. The crystal structure of the PU.1 ETS domain was sourced from PDB, id 1PUE. The raw data from Microarray have been deposited into NCBI GEO under the accession number: GSE27873.

## Code availability

The code used in this study to generate figures is available upon request.
