# Supplementary material for: PU.1-c-Jun interaction is crucial for PU.1 function in myeloid development
Source: Commun Biol. 2022 Sep 14;5:961. doi: 10.1038/s42003-022-03888-7 (PMC9474506; doi:10.1038/s42003-022-03888-7)

Supplemental Information

Methods

Densitometry quantification of the EMSA

The band density was analyzed using ImageQuant and the analysis repeated in triplicate. The value of Y-axis was calculated as follows: PU.1 complex density) / (PU.1 complex density + free probe density) x 100.

Yeast split-hybrid screen

Sequences encoding the PEST and DNA binding domains (amino acids 100-272 (Klemsz et al. 1990; Kodandapani et al. 1996)) of human PU.1 were PCR amplified with oligonucleotides engineered with BamHI (5') and KpnI (3') sites. The PU.1 fragment was then subcloned into the BamHI-KpnI sites of the pVP16 plasmid in-frame between the VP16 activation domain and the β-galactosidase gene and was used as wild-type bait. A library of PU.1 mutants was produced by random PCR mutagenesis using the oligonucleotides mentioned above, Taq polymerase, unequal concentrations of dNTPs, and inclusion of 100 μM MnCl2 in reaction buffer as described previously (Uppaluri and Towle 1995). These fragments were subcloned into pVP16, resulting colonies were pooled, and plasmids were isolated to create a library. The sequences encoding the basic domain and leucine zipper of human c-Jun (amino acids 251-331, corresponding to GenBank accession P05412) were subcloned in-frame to the DNA binding domain of lexA in pBTM116. Plasmids were introduced by sequential transformation (pBTM116-c-Jun followed by the pVP16-PU.1 mutant library) into the yeast split-hybrid strain YI671 (a generous gift from Richard Goodman) according to standard protocols. Colonies were grown on Trp-, Leu-, His- selective drop out media supplemented with 5mM 3-AT. The yeast strain Y190 was used for standard two-hybrid analysis (Zhang et al. 1999). PU.1 wild-type and mutant baits isolated from the split-hybrid yeast were transformed along with c-Jun sequences (amino acids 251-331) fused to the DNA binding domain of Gal4 in the plasmid pGBT9 (Clontech). These co-transformants were plated on Trp-, Leu-, His- media and screened for growth.

Co-immunoprecipitations and Western Blotting Analysis

For the interaction screen by co-immunoprecipitation, truncated PU.1 wild-type and mutants without the N-terminal transactivation domain was cloned into pcDNA6 and transiently transfected into Cos-7 cells with pSV1-SPORT-c-Jun using Lipofectamine and Plus Reagent (Invitrogen). 24 hours after transfection, cells were harvested, washed in PBS and lysed in 150mM NaCl, 50mM Tris pH 7.6, 1mM EDTA, 0.1% NP-40, 1mM PMSF, 1μg/μl leupeptin, 1μg/μl aprotinin, and 5% glycerol by incubating on ice for 15 minutes and passed 3 times through a 28-gauge syringe. After centrifugation at 16,000 x g for 10 minutes at 4°C, the lysate was collected and pre-cleared by incubation with 30μl blocked protein A agarose beads (Santa Cruz) and 10μg normal rabbit serum (Santa Cruz) for 1 hour at 4°C. After centrifugation to remove beads, the lysate was split into two samples and incubated with 5 μg of either normal rabbit IgG or PU.1 antibody (Santa Cruz sc-352) for 2 hours at 4°C. Then, 30μl of packed protein A agarose beads were added to each lysate and incubated overnight at 4°C. The beads were washed twice with lysis buffer, resuspended in 30μl SDS sample buffer, loaded on a 10% SDS-PAGE gel and processed for Western transfer. Western blots were probed with either anti-c-Jun (Santa Cruz sc-1694) or anti-PU.1 (sc-352) antibodies at 1:2000 dilution in TBS with 0.1% Tween-20 and 5% skim milk, incubated with anti-rabbit HRP-conjugated secondary antibody and developed with chemical-luminescent reagents (Perkin-Elmer).

qPCR Validation of Microarray

Total RNAs of both WT and KI GMP (c-kit$^+$ Sca-1$^-$ CD16/32^{hi} CD34$^+$) and ST-HSC (CD48$^-$/CD150$^-$ LSK) from murine E14.5-16.5 fetal livers were extracted with the TRIzol reagent (Invitrogen, USA). cDNAs were then synthesized using QuantiTect® Reverse Transcription Kit (RT) (QIAGEN, Germany) for RT-qPCR analysis. Quantitative real-time PCR (qPCR) analysis of the cDNAs was carried out with the iQ™ SYBR® Green Supermix (Bio-Red, USA) and the products were detected by the Rotor-Gene 6000 real-time PCR machine (Corbett Research QIAGEN, Germany). The sequences of the primers used for the qPCR analysis were home-designed and tested in Raw and A20 cell line, as shown Supplementary Table1.

Tables

Supplemental Table S1. Primers Used in qPCR Validation of Microarray Data Relating to Supplemental Figure S5

Name	Sequence
Ctss F	GCATAGAGGCAGACGCTTCCTA
Ctss R	CCACTGCTTCTTTCAGGGCATC
Ptprc F	CTTCAGTGGTCCCATTGTGGTG
Ptprc R	TCAGACACCTCTGTCGCCTTAG
Lyz F	TACAACCGTGGAGACCGAAGCA
Lyz R	TGGCTGCAGTGATGTCATCCTG
Mpeg1 F	CTCAGACCAGTTTGACGAAGGAC
Mpeg1 R	AGCACGGTCTATTGCCACTAGG
Mef2c F	GTGGTTTCCGTAGCAACTCCTAC
Mef2c R	GGCAGTGTTGAAGCCAGACAGA
Cd27 F	ATACCAGGCACCTCCTTCTCTC
Cd27 R	CACTGCCAGTTCTTGGAACAGC
Nfam1 F	GCTTTACCAGCCTGATGAGCGT
Nfam1 R	GATTCTGCTGGAGGCTTTGTGG
Irf8 F	AGACCTCATCTCCCTCCTCTCA
Irf8 R	ATAGTCACATCCAGAGCCAGCA
Alcam F	AAGTATTCCAGAGCACGATGAGG
Alcam R	GGAGACCAACGACAATTCCCA
Ptprf F	GACCACCTACTCCATCACCG
Ptprf R	GTGGTCAGTCCTGTCACTCG
Gfi1b F	CCGCCTGCTTTAGTGTGTCT
Gfi1b R	CGGATGGATGTGGTGAGAGG
Trat1 F	CCCGCTAGACTGTTTGGGTT
Trat1 R	TGAAGCTCAGGATGGAGCAC
Adamts1 F	TTGAATGGTGTGAGTGGCGA
Adamts1 R	TTGGATTCTGGGGCTTGTCC

Figures

Supplementary Fig S1:

Identification of PU.1 mutants by yeast split-hybrid assay.

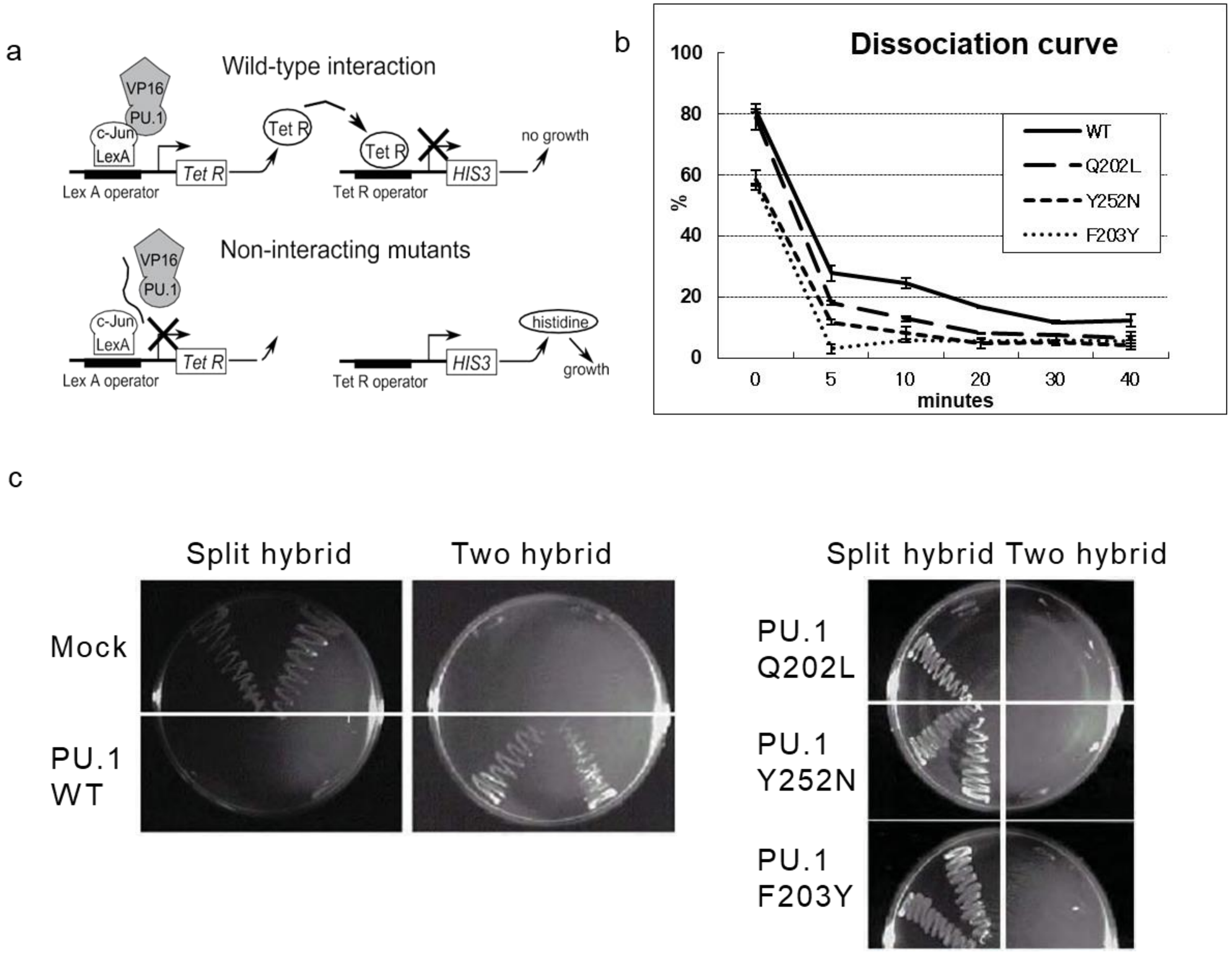

(a) Schematic of the screening system. Interacting proteins are fused separately to the LexA DNA binding domain (DBD) and to the VP16 transactivation domain, respectively. Interaction leads to the transcription of the tetracycline repressor (TetR) in the split-hybrid strain YI671, which can subsequently bind to a TetR site upstream of the HIS3 gene, thereby preventing growth in the absence of exogenous histidine. Non-interacting proteins do not synthesize TetR, allowing the yeast strain to synthesize histidine and to grow on histidine-free medium. The region of human c-Jun interacting with PU.1 (amino acids 251-334) attached to the LexA DBD was used as a bait, and the mutant human PU.1 library (containing amino acids 100-272 encompassing PEST and Ets domains) was cloned in-frame with the VP16 transactivation domain. (b) The densitometry quantification of Fig. 1b. (b) Identification of three-point mutations by the split-hybrid assay. Vector control ("mock") and PU.1 wild-type and mutant vectors were used in yeast split-hybrid and twohybrid assay. The right panels show PU.1 mutants which score in the split-hybrid but not in the twohybrid assay, demonstrating that these

mutants fail to interact with the c-Jun bait. Standard two-hybrid analysis allows growth upon interaction of bait and library and growth inhibition when no interaction is taking place.

Supplementary Fig. S2:

PU.1 mutants localize to the nucleus.

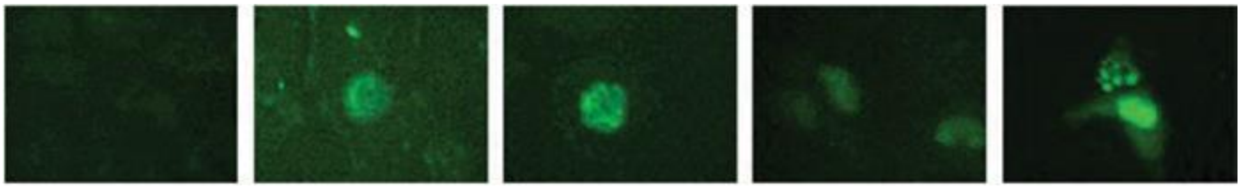

Cos7 cells transfected with wild-type or mutant PU.1 expression vectors were stained with anti-PU.1 antiserum and analyzed by immunofluorescence microscopy. Wild-type and mutants localized equally efficiently in the nucleus, demonstrating that the failure of these mutants to transactivate their targets is not a result of loss of a nuclear localization function. Assay was repeated 3 times with representative blots shown.

Supplementary Fig. S3:

PU.1 mutant is expressed in hematopoietic stem and progenitor cells.

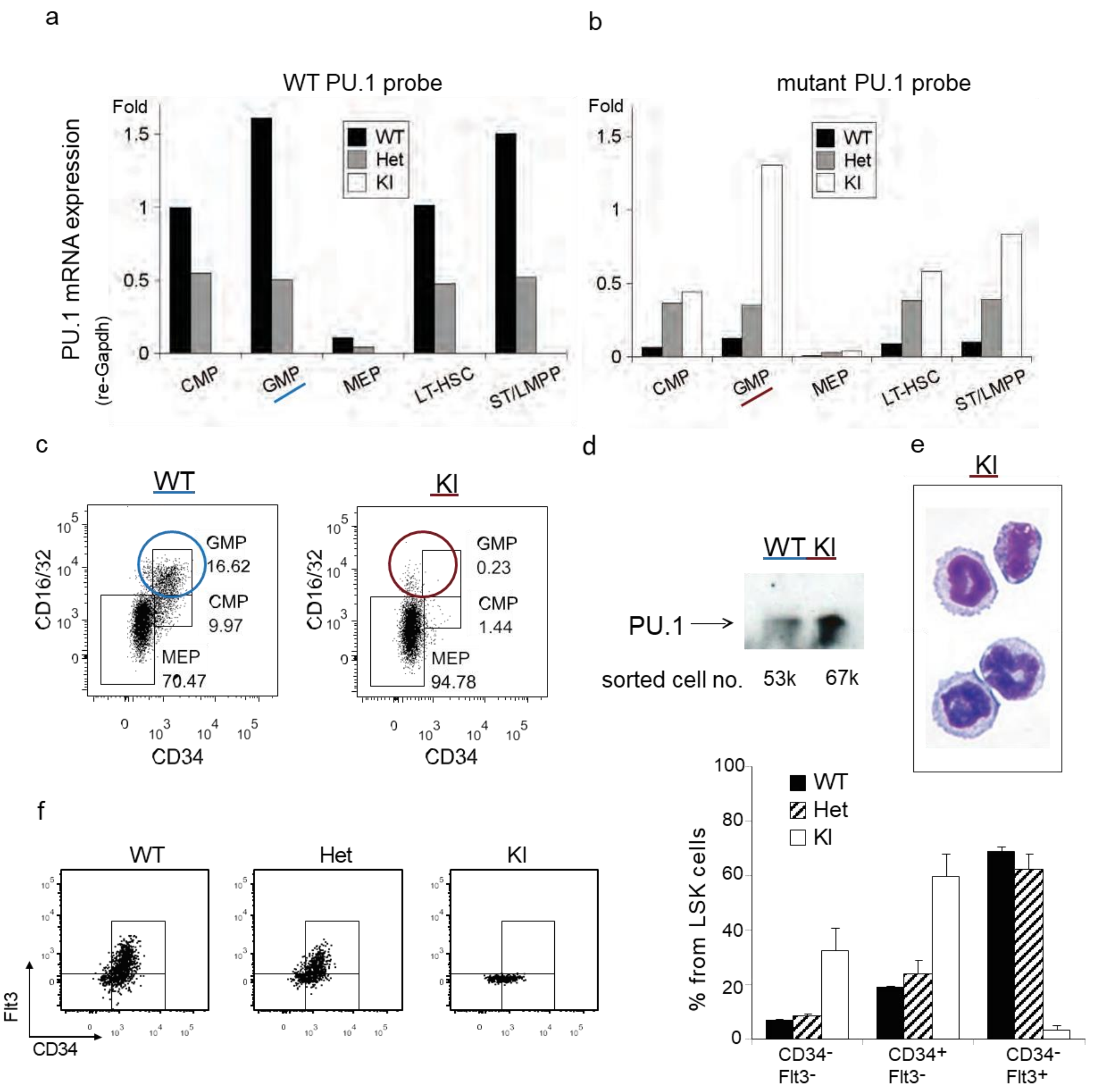

Hematopoietic stem and progenitor cell populations from E14.5-16.5 fetal liver (FL) cells were sorted by flow cytometry and mRNA was isolated. LT-HSC were defined as lin- ($CD4^-$ $CD8^-$ $B220^-$ $CD19^-$ $Gr1^-$) c-kit^+ Sca-1^+ $CD34^-$ $Flt3^-$ cells, ST-HSC/LMPP as lin- c-kit^+ Sca-1^+ $CD34^+$ cells. PU.1 mRNA levels were measured separately for wild-type (a) and mutant cells (b) in wild-type (WT, black bars), heterozygous (Het, grey bars), and homozygous mutants (KI, white bars) by quantitative real-time PCR using TaqMan MGB probes distinguishing between the two genotypes. (c). Representative

FACS diagram of lin$^-$ c-kit$^+$ Sca-1$^-$ progenitor cells from WT and KI E14.5-16.5 FL. WT GMP and the KI GMP-like cells are indicated by blue and red circles, respectively. (d) Western blot analysis of sorted cells of WT GMP and the GMP-like population (encircled in C). The sorted cell number of each population is indicated. (e) Wright-Giemsa-stained sorted CD34^{lo} CD16/32^{hi} (FcγRII/III^{hi}) population of cells resembling immature myeloid cells (population encircled in red in (c)) is shown. (f) Flow cytometry analysis of E16.5 fetal liver cells. The relative contribution of the listed cell populations to total live (DAPI -) fetal liver cells is shown as the mean of values from quadruplicates ± SD. Representative FACS profiles are shown for wildtype (WT), heterozygous (Het) and homozygous PU.1 Q202L knock-in cells (KI). Experiments were performed at least three times with similar results. Analysis of hematopoietic KSL (c-kit+ + Sca-1+ lin-) cells. For the data presented herein, three independent experiments were performed with representative results shown.

Supplementary Fig. S4:

Mutant PU.1 remains capable of binding other known interaction partners in coimmunoprecipitation assays.

a

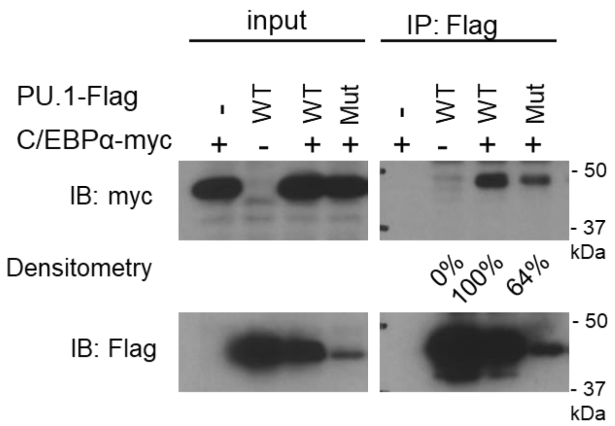

b

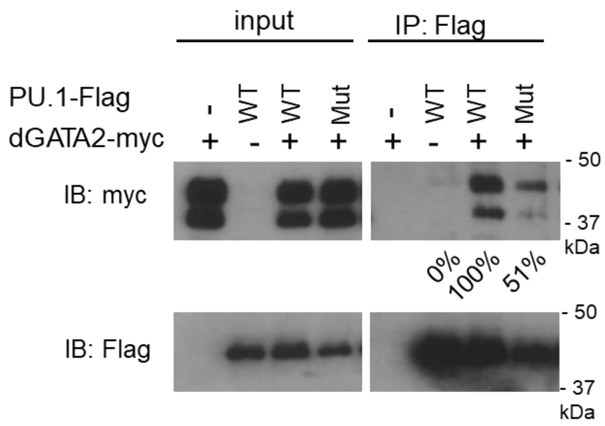

Flag-tagged human PU.1 wild-type (WT) or Q202L mutant (Mut) were expressed in 293T cells together with human myc-tagged C/EBPα (a) or drosophila GATA-2 (b), and immunoprecipitated with anti-Flag antibody. The precipitate was immunoblotted (IB) with an antibody recognizing the myc tag (top panels) and the Flag tag (lower panels), respectively. For panel (a), densiometric analysis of protein levels shows that in the Flag IP, the Mut is still capable of pulling down 64% of the WT PU.1 with C/EBPα-myc protein levels. Likewise for panel (b), the Mut is capable of pulling down over 50% of the WT PU.1 with dGATA2-myc. Three independent experiments were performed with representative results shown.

Supplementary Fig. S5:

PU.1 mutant is expressed in hematopoietic stem and progenitor cells.

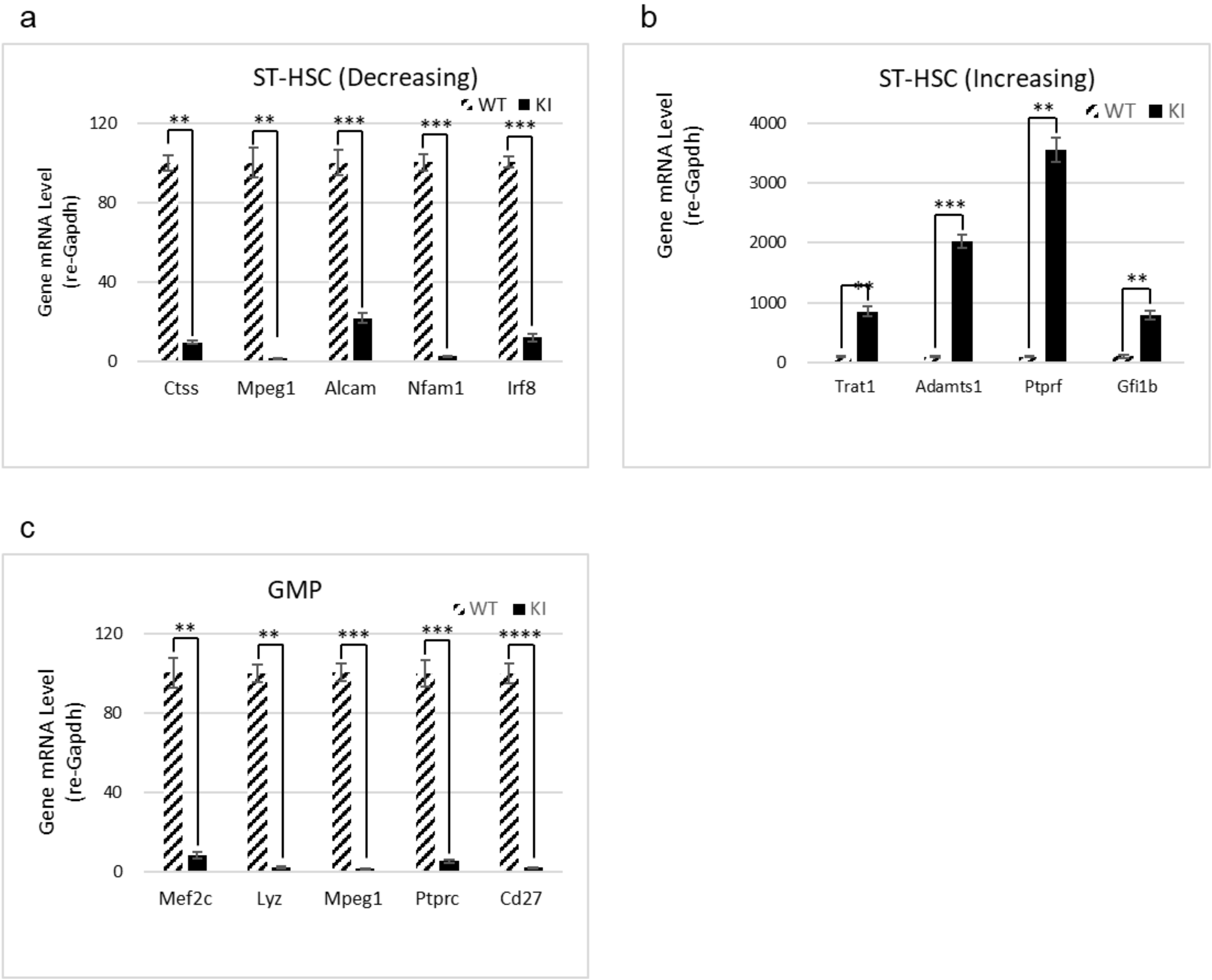

ST-HSC (CD150$^-$ CD48$^-$ LSK) and GMP (lin$^-$ ckit$^+$Sca1$^-$ CD16/32^{hi} CD34$^+$) from E14.5-16.5 fetal liver cells of both WT and KI embryos (both genders) were sorted by flow cytometry and mRNA was isolated. Different genes indicated in microarray (Fig. 5a) mRNA levels were measured separately for ST-HSC (decreasing) (a) and ST-HSC (increasing) (b) and GMP (decreasing) (c), wild-type (WT, slash bars) and homozygous mutants (KI, black bars) by qPCR using iQTM SYBR® Green. Primers utilized can be found listed in Supplemental Table S1. Three independent experiments were performed. Experiment details in Supplemental Methods. All statistical comparisons are WT vs KI samples and p-values calculated using two-tailed t-test with Welch's Correction. * $p<0.05$, ** $p<0.01$, *** $p<0.001$ **** $p<0.0001$.

Supplementary Fig. S6:

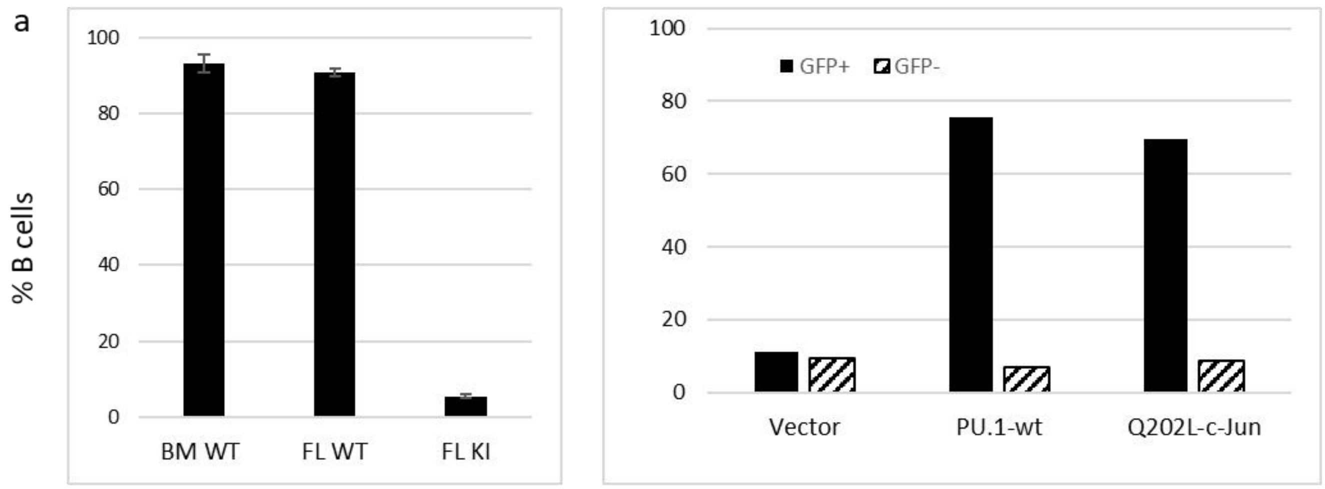

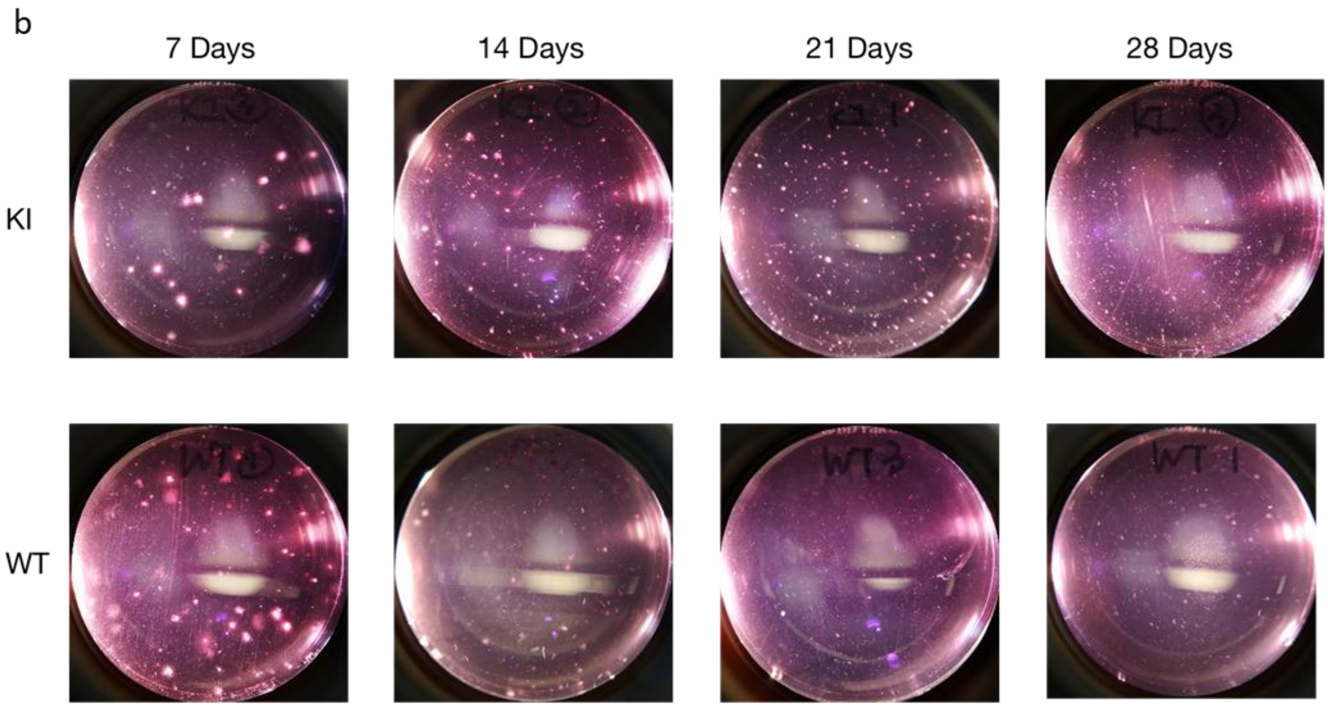

(a) The bar graph quantification of Fig. 4d (left) and 4e (right). (b) Colonies of both KI and WT in 35mm plate under 0.63X microscope. Relative to Fig. 3c. Multiple independent plates were cultured (at least n=3), representative figures shown.

Supplementary Fig. S7

a) Raw blots for Figure 1b

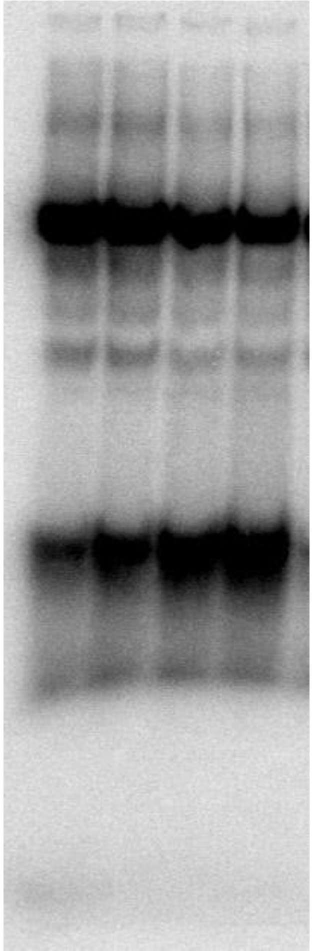

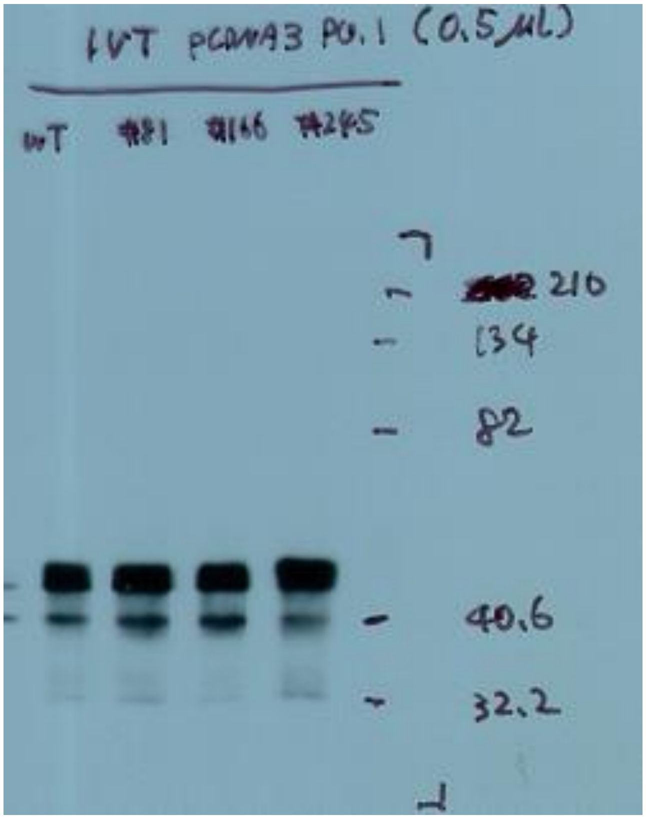

Wild-type PU.1 Q202L Y252N F203Y

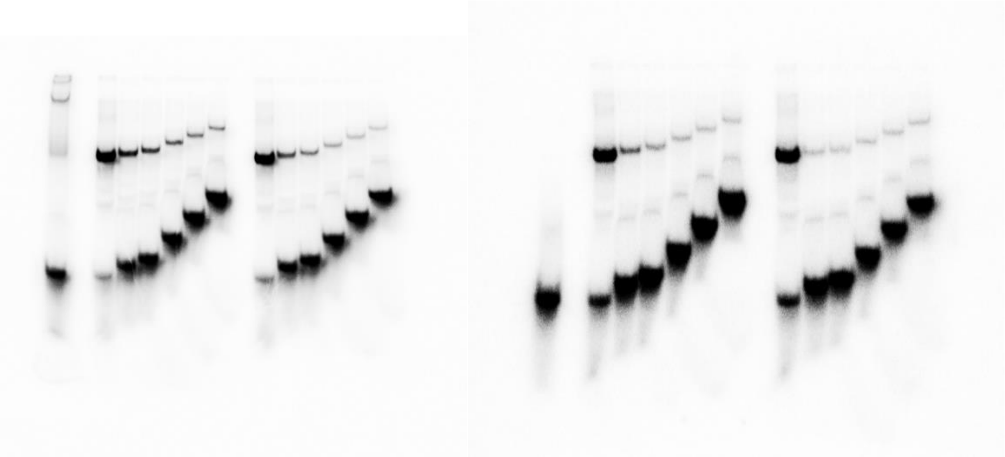

b) Raw blots for Figure 1c

Wild-type

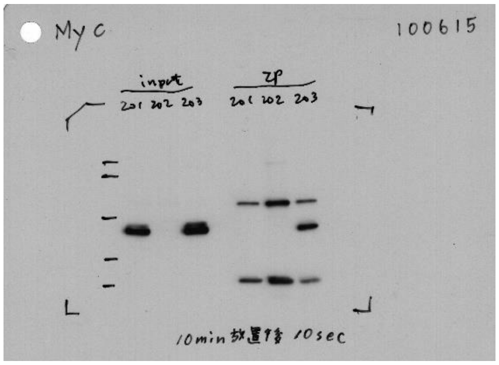

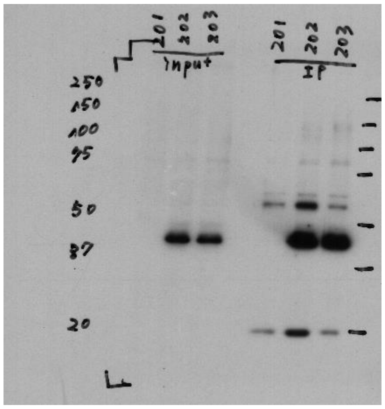

Q202L

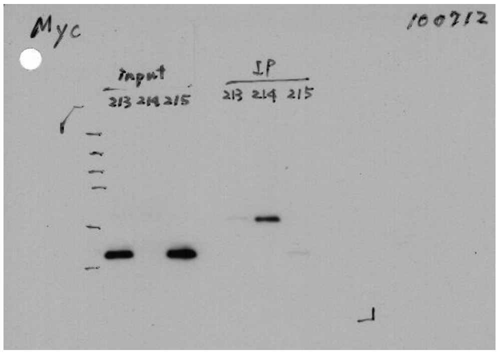

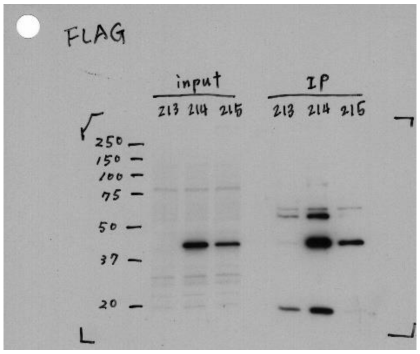

Y252N

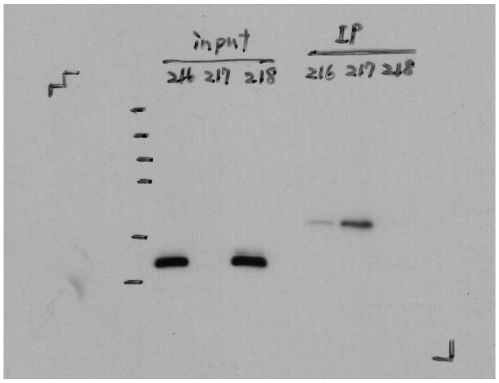

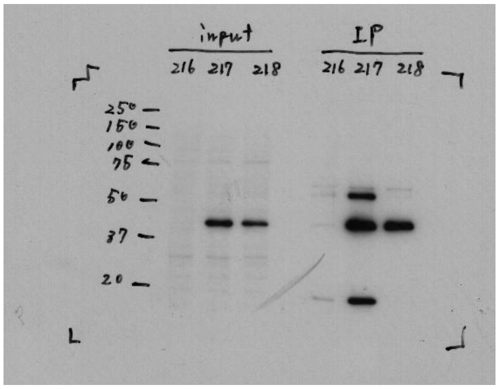

F203Y

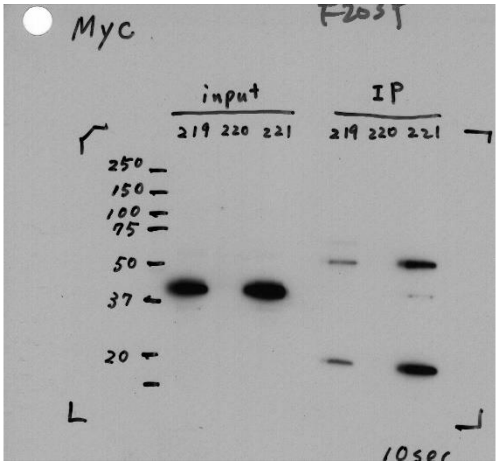

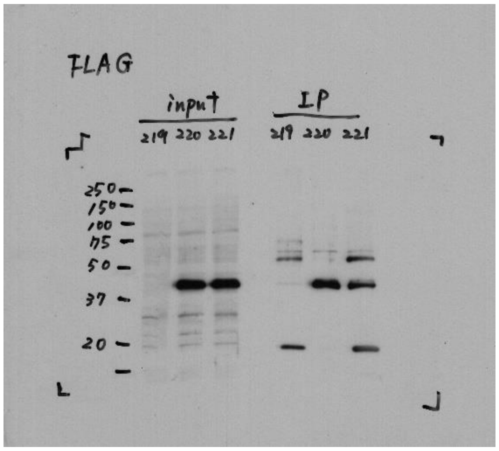

c) Raw blots for Figure 2c

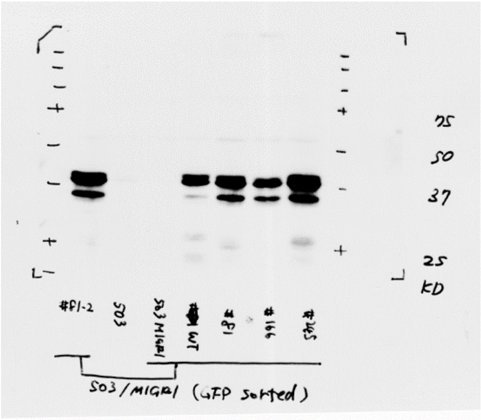

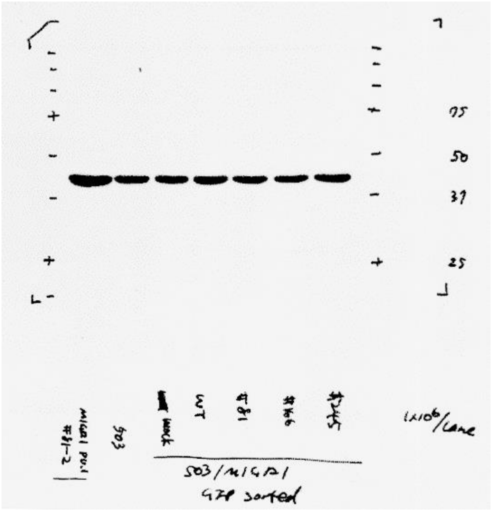

d) Raw blots for Supplemental Figure 4a

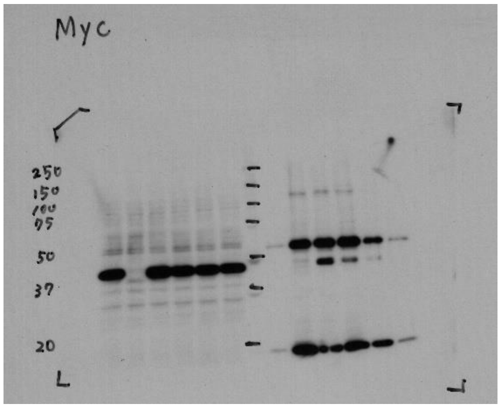

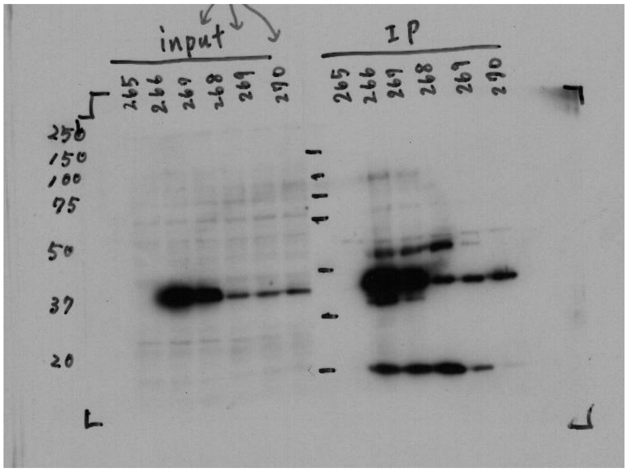

e) Raw blot for Supplemental Figure 4b

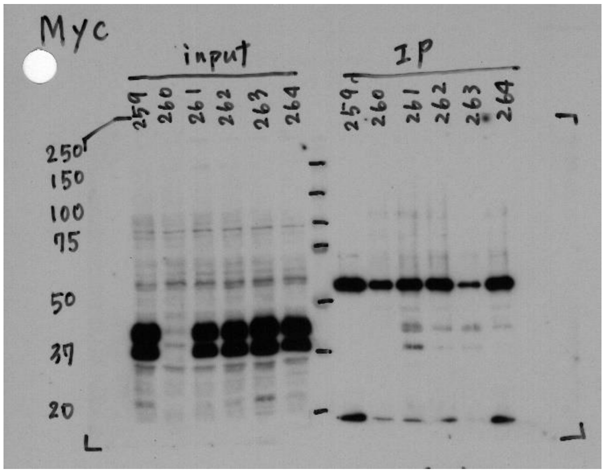

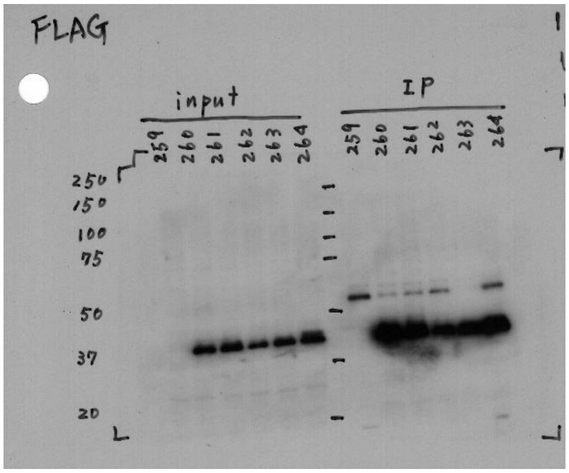

Supplement: Supplementary file 1 — Supplemental Information [file 42003_2022_3888_MOESM1_ESM.pdf]
