# Supplementary material for: PU.1-c-Jun interaction is crucial for PU.1 function in myeloid development
Source: Commun Biol. 2022 Sep 14;5:961. doi: 10.1038/s42003-022-03888-7 (PMC9474506; doi:10.1038/s42003-022-03888-7)
Supplement: Supplementary file 2 — Description of Additional Supplementary Files [file 42003_2022_3888_MOESM2_ESM.pdf]

Description of Additional Supplementary Files

File name: Supplementary Data 1
Description: Numerical source data behind main figure graphs.
